# A Conserved Carbon Starvation Response Underlies Bud Dormancy in Woody and Herbaceous Species

**DOI:** 10.3389/fpls.2017.00788

**Published:** 2017-05-23

**Authors:** Carlos Tarancón, Eduardo González-Grandío, Juan C. Oliveros, Michael Nicolas, Pilar Cubas

**Affiliations:** ^1^Plant Molecular Genetics Department, Centro Nacional de Biotecnología (Consejo Superior de Investigaciones Científicas), Campus Universidad Autónoma de MadridMadrid, Spain; ^2^Bioinformatics for Genomics and Proteomics Unit, Centro Nacional de Biotecnología (Consejo Superior de Investigaciones Científicas), Campus Universidad Autónoma de MadridMadrid, Spain

**Keywords:** bud dormancy, carbon starvation, gene regulatory networks, shoot architecture, plant evolution and development

## Abstract

Plant shoot systems give rise to characteristic above-ground plant architectures. Shoots are formed from axillary meristems and buds, whose growth and development is modulated by systemic and local signals. These cues convey information about nutrient and water availability, light quality, sink/source organ activity and other variables that determine the timeliness and competence to maintain development of new shoots. This information is translated into a local response, in meristems and buds, of growth or quiescence. Although some key genes involved in the onset of bud latency have been identified, the gene regulatory networks (GRNs) controlled by these genes are not well defined. Moreover, it has not been determined whether bud dormancy induced by environmental cues, such as a low red-to-far-red light ratio, shares genetic mechanisms with bud latency induced by other causes, such as apical dominance or a short-day photoperiod. Furthermore, the evolution and conservation of these GRNs throughout angiosperms is not well established. We have reanalyzed public transcriptomic datasets that compare quiescent and active axillary buds of Arabidopsis, with datasets of axillary buds of the woody species *Vitis vinifera* (grapevine) and apical buds of *Populus tremula x Populus alba* (poplar) during the bud growth-to-dormancy transition. Our aim was to identify potentially common GRNs induced during the process that leads to bud para-, eco- and endodormancy. In Arabidopsis buds that are entering eco- or paradormancy, we have identified four induced interrelated GRNs that correspond to a carbon (C) starvation syndrome, typical of tissues undergoing low C supply. This response is also detectable in poplar and grapevine buds before and during the transition to dormancy. In all eukaryotes, C-limiting conditions are coupled to growth arrest and latency like that observed in dormant axillary buds. Bud dormancy might thus be partly a consequence of the underlying C starvation syndrome triggered by environmental and endogenous cues that anticipate or signal conditions unfavorable for sustained shoot growth.

## Introduction

Shoot branching patterns define overall above-ground plant architecture. In angiosperms, shoots are formed from axillary meristems initiated at the base of leaves. These meristems grow and develop into axillary buds that contain, preformed, most of the elements of adult branches (shoot meristems, leaf primordia, reproductive meristems). Axillary buds can enter a quiescent state, rather than growing out immediately to give a branch. In this latent or dormant state their metabolic activity and cell division are very limited (Shimizu and Mori, 1998; Ruttink et al., 2007). Bud dormancy and bud activation are influenced by environmental signals such as nutrient and water availability, light quality, day-length and temperature, and by endogenous signals such as sink/source organ activity and hormone signaling. Once dormant, buds require changes in specific developmental and/or environmental cues to resume growth and generate an elongated branch. These cues are monitored in different organs, and inform the plant as to when develop new shoots. This information is transduced to the bud and translated into a gene response that leads to quiescence or growth activation (Rameau et al., 2015).

Bud dormancy is therefore an adaptive trait that allows plants to endure adverse situations until conditions are favorable for development of new shoots. It has great impact on plant reproductive success and productivity, and on survival in temperate woody species. Evolution of this trait might have allowed plants to colonize habitats with fluctuating conditions not always suitable for sustained, uninterrupted growth. Depending on the type of stimulus that promotes growth arrest, Lang et al. (1985, 1987) distinguished three types of bud dormancy. When dormancy is induced by environmental factors, it is termed *ecodormancy*; when promoted by other plant organs it is *paradormancy* or correlative inhibition, and when it is maintained by signals internal to the bud and can only be reversed under certain conditions it is defined as *endodormancy*. In woody plants, axillary buds undergo transitions between different dormant states throughout the year. Paradormant buds enter endodormancy in response to changes in daylength and temperature. Chilling promotes transition from endo- to ecodormancy, after which the buds are susceptible to grow in response to mild temperatures (Rohde and Bhalerao, 2007).

Transcriptomic studies have been carried out in several herbaceous and woody species to define expression changes in buds during the transitions into and out of different types of dormancy, in response to changes in daylength, light quality, and apical dominance, and in mutant genotypes in which bud growth is affected (e.g., Tatematsu et al., 2005; Ruttink et al., 2007; González-Grandío et al., 2013; Reddy et al., 2013; Ueno et al., 2013; Porto et al., 2015). The GRNs that act inside the bud to control the stages leading to dormancy nonetheless remain little known. It is also largely unknown whether different types of dormancy share common underlying genetic mechanisms. Even less is known about the degree of conservation and evolution of the genetic control of this process in different plant species. Comparative analyses to identify common themes among different types of dormancy, or across species, are scarce (González-Grandío and Cubas, 2014; Fennell et al., 2015; Howe et al., 2015; Hao et al., 2017). Such comparisons could help us determine whether eco-, para- and endodormancy are variations of a single ancestral genetic program or whether each type is controlled by unrelated GRNs. It also will help elucidate whether GRNs that cause bud growth arrest are conserved in different herbaceous and woody plant species.

The master regulators that locally control the dormancy onset are also largely unknown. The best characterized are the genes that encode the TCP transcription factors (TF) teosinte branched1 (Tb1, Doebley et al., 1997), BRANCHED1 (BRC1, Aguilar-Martínez et al., 2007; Finlayson, 2007) and their orthologs in mono- and dicotyledonous species, respectively. These widely conserved factors play a very important role in the regulation of para- and ecodormancy in herbaceous plants. These genes are expressed in axillary buds and promote bud dormancy in response to fluctuating environmental cues such as light quality and quantity, and endogenous signals such as apical dominance, sugar availability and hormone signaling (reviewed in Nicolas and Cubas, 2015). In *Arabidopsis thaliana, BRC1* controls transcription of several GRNs in buds; one, positively controlled by *BRC1*, leads to abscisic acid (ABA) accumulation and signaling, whereas another two that are downregulated by *BRC1* are enriched in ribosomal protein genes in one case, and in cell division and DNA replication genes in the other (González-Grandío et al., 2013, 2017). Additional GRNs controlled by *BRC1* remain to be characterized.

In this study our aim was to identify potentially common GRNs induced during the process that leads to bud para-, eco- and endodormancy. For that we compared publicly available transcriptomic data from active para- and ecodormant axillary buds of Arabidopsis, and found, induced in dormant buds, a shared transcriptomic response typical of tissue undergoing C starvation. We then detected this response also in *Populus tremula × Populus alba* (poplar) apical buds undergoing endodormancy and in *Vitis vinifera* (grapevine) axillary buds entering para-, endo- and ecodormancy. This C starvation transcriptional response, activated shortly after exposure to conditions leading to bud dormancy, anticipates and underlies the growth-to-dormancy transition in the three species. The C starvation syndrome entails a suite of interconnected transcriptional responses that include sugar signaling, sugar metabolism reprogramming, senescence, autophagy, catabolism, and ABA and ethylene signaling. It also involves downregulation of cytokinin (CK) signaling, inhibition of anabolism, and repression of protein/DNA synthesis and cell division, conditions typical of cells in dormant buds. This conserved starvation response, genetically connected to cell growth arrest, may be one of the underlying forces driving the growth-to-dormancy transition of axillary buds in response to suboptimal conditions in herbaceous and woody species.

## Results

### Arabidopsis Bud Dormancy Is Associated With the Induction of Four GRN

Three independent transcriptomic analyses have compared active and dormant buds in Arabidopsis. One study compared the transcriptional profiling of (dormant) buds of intact plants and of (active) buds of decapitated plants at 24 h post-treatment (Tatematsu et al., 2005). Two additional experiments compared the transcripts of active vs. dormant buds of plants exposed to high red-to-far-red light ratio (R:FR, active buds) or low R:FR (dormant buds) (González-Grandío et al., 2013; Reddy et al., 2013). Here we define dormancy as a state in which bud growth is reversibly interrupted, regardless of the requirements to resume development. A search for genes upregulated in dormant buds relative to active buds in the three experiments identified 78 genes termed *bud dormancy* genes (Supplementary Figure S1 and Dataset S1; González-Grandío and Cubas, 2014). These genes correspond to the least common denominator of the three studies and were induced in para- and ecodormant buds by either correlative inhibition or low R:FR, respectively. They were also differentially expressed at 3 h (Reddy et al., 2013), 8 h (González-Grandío et al., 2013) and 24 h (Tatematsu et al., 2005) after treatment onset.

**FIGURE 1:**
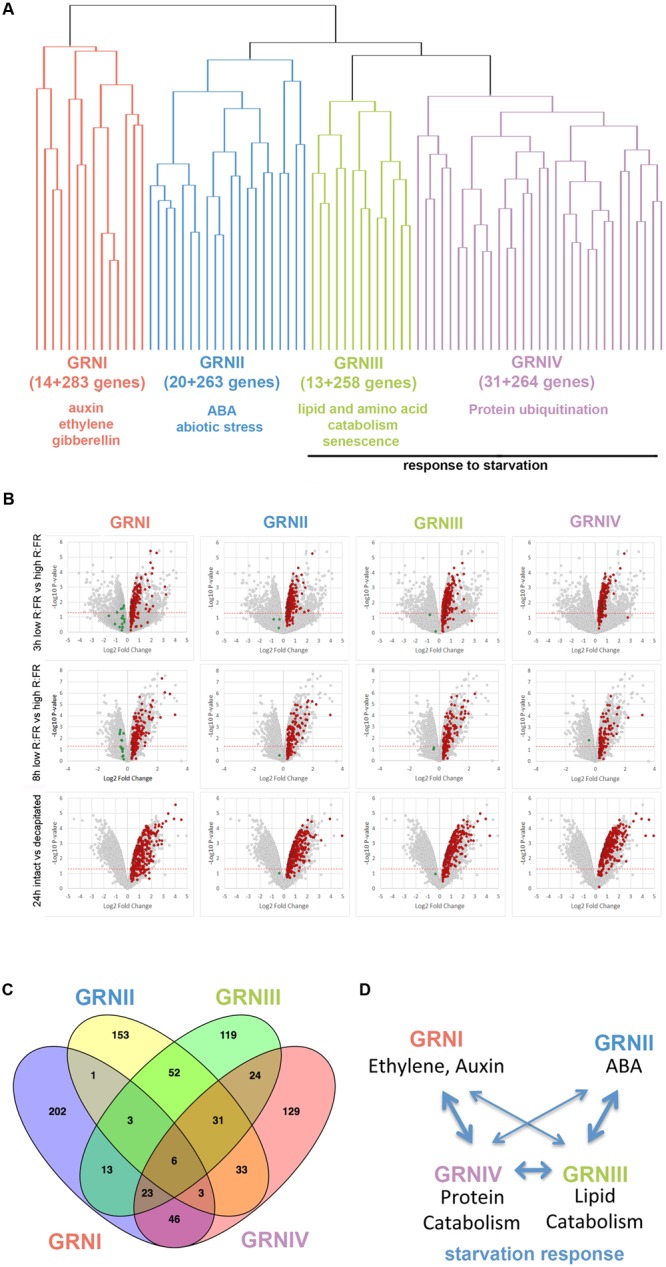
**Bud dormancy genes and GRN. (A)** Hierarchical clustering representation of bud dormancy genes (González-Grandío and Cubas, 2014) based on their degree of coregulation in 15,275 microarray experiments (ATTED-II; Obayashi et al., 2007). The number of coregulated genes and GO terms enriched are indicated. **(B)** Volcano plots representing pval (–Log10 pval, vertical axis) and relative expression (Log2 fold change, horizontal axis) of all genes in each microarray. Normalized gene intensities in dormant buds vs. normalized gene intensities in active buds were compared in all experiments [3 h low R:FR (N-2 bud) vs. high R:FR (N-2 bud); 8 h low R:FR vs. High R:FR; intact plants vs. 24 h post-decapitation]. Bud dormancy genes and their coregulated genes are highlighted. In red and green, genes induced and repressed in dormant buds, respectively. Genes highlighted in red were attributed to Bud dormancy GRNI-IV (see Supplementary Dataset S1) and were used for subsequent analyses. **(C)** Venn diagram showing overlap between bud dormancy GRN. Number of common genes is indicated. **(D)** Model of the relationships between bud dormancy GRN. Line thickness indicates degree of overlap between GRN.

We evaluated the degree of coregulation of these genes using the most updated co-expression database of ATTED-II (15,275 microarray experiments; Obayashi et al., 2007). Hierarchical clustering analysis revealed four clusters of coregulated genes (14, 20, 13, and 31 genes; **Figure 1A** and Supplementary Dataset S1). We then searched for additional genes coregulated with each cluster using CoExSearch (ATTED-II, Obayashi et al., 2007) and obtained four lists of highly coregulated genes (Supplementary Dataset S1). Analysis of their fold change (FC) induction in the three active-vs.-dormant bud experiments confirmed that a significant proportion of the genes in each list were induced (FC ≥ 1.2) in dormant buds in at least one experiment (**Figure 1B** and Supplementary Figure S2). We termed the gene lists that comprised the bud dormancy genes of the original clusters plus their coregulated genes (induced in dormant buds in at least one experiment, red dots in **Figure 1B**) *bud dormancy GRNI-IV*, with 297, 283, 271, and 295 genes respectively (Supplementary Dataset S1).

### Bud Dormancy GRNs Are Related to Hormone Signaling, Stress, Catabolism and Starvation Response

To elucidate the biological processes in which these GRNs were involved, we searched for enrichment in gene ontology (GO) terms using the Panther Classification System (Mi et al., 2017; Supplementary Dataset S2), complemented with a MapMan bin analysis (Thimm et al., 2004; Supplementary Dataset S1). GRNI was significantly enriched in terms related to ethylene, auxin and gibberellin signaling and response; GRNII in terms related to ABA, catabolism and response to abiotic stress; GRNIII in terms related to lipid and amino acid catabolism, senescence, response to starvation and biotic stress; and GRNIV in terms related to protein ubiquitination and response to sucrose starvation.

We evaluated the degree of overlap between these GRNs by seeking common genes. GRNIII and GRNIV shared one-third of their genes; GRNII and GRNIII shared 30%, and GRNI and GRNIV had 26% genes in common (**Figure 1C**, Supplementary Figure S3 and Dataset S3). This suggested that these GRNs are not strictly independent, but correspond to related aspects of the same syndrome, probably coordinated or maintained by ethylene, auxin and ABA signaling (**Figure 1D**).

### Bud Dormancy GRNs Are Enriched in Genes Typical of a C Starvation Response

We observed that three robust sugar starvation gene markers, *GIBBERELLIN-STIMULATED ARABIDOPSIS 6* (*GASA6*), *DORMANCY-ASSOCIATED PROTEIN-LIKE 1* (*DRM1/DYL1*) and *DARK INDUCIBLE 6* (*DIN6*) (Contento et al., 2004; Price et al., 2004; Gonzali et al., 2006; Zhong et al., 2015) were members of one or several GRNs (*GASA6*, GRNI; *DRM1*, GRNI, III and IV; *DIN6*, GRNIII and IV; Supplementary Dataset S3). As sugar has a prominent role in the control of shoot outgrowth (Mason et al., 2014; Barbier F. et al., 2015; reviewed in Barbier F.F. et al., 2015) and GRNIV was significantly enriched in terms related to sucrose starvation, we studied this response further. The C starvation syndrome, triggered under C-limiting conditions (e.g., an extended night), helps to obtain an alternative energy source and C skeletons. In Arabidopsis, it comprises a suite of interconnected events that result in changes in C balance and growth. They include reprogramming of sugar sensing, transport, signaling and metabolism, increased protein ubiquitination and degradation, amino acid and lipid catabolism, induction of ABA and ethylene signaling, and recycling of cell components via autophagy and senescence. In addition, CK signaling, ribosomal gene expression, DNA synthesis and cell division are inhibited (Contento et al., 2004; Lin and Wu, 2004; Thimm et al., 2004; Gonzali et al., 2006; Rolland et al., 2006; Rose et al., 2006). Remarkably, many of the GO terms and/or MapMan bins enriched in the four GRNs matched categories induced by C-limiting conditions (**Table 1** and Supplementary Datasets S1, S2).

**Table 1:** Bud dormancy genes from categories related to sugar sensing, transport, signaling and metabolism, protein ubiquitination and degradation, as well as amino acid and lipid catabolism, autophagy and senescence.

AGI	Name	GRN	AGI	Name	GRN
**Sugar**			**Senescence**		
Transport			At4g37790	*ABA INSENSITIVE GROWTH 1 (ABIG1/HAT22)*	ll-lll
At3g48740	*SUCROSE EFLUX TRANSPORTER SWEET11*	I	At1g01720	*ATAF1*	II
At5g23660	*SUCROSE EFFLUX TRANSPORTER SWEET12*	I	At1g69490	*NAP*	I-II-III-IV
At2g43330	*INOSITOL TRANSPORTER 1*	II	At5g39610	*ORE1*	I-II-III
At1g22710	*SUCROSE TRANSPORTER 1 (SUT1/SUC2)*	I	At5g51070	*SAG15*	III
At1g11260	*STP1*	I-lll	At3g10985	*SAG20*	III-IV
At5g61520	*STP3*	I	At4g02380	*SAG21*	III
At1g77210	*STP14*	I-lll	At5g59220	*SAG113/HAI1*	ll
At1g08920	*SUGAR TRANSPORTER ERD6-LIKE 3*	II	At4g35770	*SEN1/DIN1*	I-III-IV
			At1g62300	*WRKY6*	III
Sensing			At2g42620	*ORE9/MAX2*	II-IV
At5g64260	*EXL2*	II-III-IV	**Autophagy**		
At5g09440	*EXL4*	ii	At3g51840	*ATG6*	IV
			At4g21980	*ATG8A*	III
Signaling			At4g04620	*ATG8B*	III
At5g21170	Snrkl submit β1 *(AKINBETA1)*	IV	At1g62040	*ATG8C*	IV
At3g48530	Snrkl subunit γ1 *(KING1)*	II-III-IV	At2g45170	*ATG8E*	I-III-IV
At1g68020	*TPS6*	II	At4g16520	*ATG8F*	I-IV
At1g70290	*TPS8*	III-IV	At3g06420	*ATG8H*	II
At1g23870	*TPS9*	III-IV	At3g15580	*ATG81*	IV
At1g60140	*TPS10*	II-III-IV	At1g54210	*ATG12A*	IV
At2g18700	*TPS11*	II-III-IV	At1g54710	*ATG18H*	II
At4g24040	*TREHALASE*	III	At3g62770	*ATG18A*	III
			At5g54730	*ATG18F*	II-III
**Metabolism**					
At1g13700	*6-PHOSPHOGLUCONOLACTONASE 1*	IV	**Lipid degradation**		
At1g54100	*ALDEHYDE DEHYDROGENASE H7B4*	II-III	At4g16760	*ACYL-COENZYME A OXIDASE 1 (ACX1)*	III
At3g23920	β*-AMYLASE 1 (BAM 1)*	II	At5g65110	*ACX2*	III
At5g55700	*BAM 4*	II-III	At3g51840	*ACX4*	IV
At2g42790	*CITRATE SYNTHASE 3*	II-III	At1g68620	*CARBOXYLESTERASE 6*	II-III
At2g47180	*GALACTINOL SYNTHASE 1 (GOLS 1)*	II	At1g48320	*1,4-D1HYDROXY-2-NAPHTHOYL-COA*	III
At1g56600	*GOLS 2*	II		*THIOESTERASE 1*	
At1g08940	*PHOSPHOGLYCERATE MUTASE AT74H*	III	At5g18640	α/β-Hydrolases superfamily protein	I-IV
At4g15530	*PYRUVATE, PHOSPHATE DIKINASE 1*	III	At2g39400	α/β-Hydrolases superfamily protein	I-III-IV
At5g51970	*SORBITOL DEHYDROGENASE*	IV	At1g73920	α/β-Hydrolases superfamily protein	II
At4g02280	*SUCROSE SYNTHASE 3*	II	At1g18460	α/β-Hydrolases superfamily protein	II
			At5g16120	α/β-Hydrolases superfamily protein	III
			At5g18630	α/β-Hydrolases superfamily protein	IV
			At3g60340	α/β-Hydrolases superfamily protein	IV
			**Aminoacid degradation**		
			At1g55510	*2-OXOISOVALERATE DEHYDROGENASE* β1	IV
			At4g33150	*α-AMINOADIPIC SEMIALDEHYDE SYNTHASE*	II-III
			At5g53970	*AMINOTRANSFERASE TAT2*	II
			At5g54080	*HOMOGENTISATE 1,2-DlOXYGENASE*	II-III-IV
			At3g45300	*ISOVALERYL-COA DEHYDROGENASE*	II-III-IV
			At1g64660	*METHIONINE GAMMA-LYASE*	II-III
			At4g34030	*METHYLCROTONOYL-COA CARBOXYLASE* β	IV
			At1g03090	*METHYLCROTONOYL-COA CARBOXYLASE α*	III-IV
			At1g08630	*THREONINE ALDOLASE 1*	III
**Protein degradation**					
At5g57360	*ADAGIO 1*	II	At1g80440	*KMD1*	I-III-IV
At2g18915	*ADAGIO 2*	II	At1g15670	*KMD2*	III
At1g05840	*ASPARTYL PROTEASE*	IV	At2g44130	*KMD3*	I-III-IV
At1g21780	BTB/POZ domain-containing protein	II-IV	At3g59940	*KMD4/SKIP20*	I-IV
At5g18650	CHY-/CTCHY-/RING-type Zinc finger protein	IV	At4g24990	*ATGP4*	III
At5g22920	CHY-/CTCHY-/RING-type Zinc finger protein	IV	At1g23440	*PEPTIDASE C15*	III
At3g13550	*COP10*	IV	At4g02440	*PHYTOCHROME A-ASSOCIATED*	IV
At2g40880	Cysteine proteinase inhibitor 3	I	At1g60190	*PLANT U-BOX 19*	II
At5g05110	Cysteine proteinase inhibitor 7	II	At2g22690	RING finger protein	II
At4g39090	Cysteine proteinase RD19a	IV	At1g13195	RING/U-box superfamily protein	III
At5g04250	Cysteine proteinases superfamily protein	II	At1g14200	RING/U-box superfamily protein	II
At4g23450	E3 ubiquitin ligase	II	At1g24440	RING/U-box superfamily protein	II-IV
At5g42200	E3 ubiquitin ligase ATL23	IV	At1g26800	RING/U-box superfamily protein	II-IV
At1g74410	E3 ubiquitin ligase ATL24	II	At1g49850	RING/U-box superfamily protein	IV
At3g05200	E3 ubiquitin ligase ATL6	III	At1g55530	RING/U-box superfamily protein	II
At1g49210	E3 ubiquitin ligase ATL76	I	At1g75400	RING/U-box superfamily protein	II
At1g76410	E3 ubiquitin ligase ATL8	I-III-IV	At2g15580	RING/U-box superfamily protein	I
At3g09770	E3 ubiquitin ligase LOG2	II	At2g37150	RING/U-box superfamily protein	II
At4g11360	E3 ubiquitin ligase RHA1B	I-III-IV	At3g02340	RING/U-box superfamily protein	II-III-IV
At5g22000	E3 ubiquitin-protein ligase RHF2A	II-III	At3g05250	RING/U-box superfamily protein	IV
At4g03510	E3 ubiquitin-protein ligase RMA1	I-III-IV	At3g13430	RING/U-box superfamily protein	III
At4g28270	E3 ubiquitin-protein ligase RMA2	IV	At3g15070	RING/U-box superfamily protein	IV
At4g27470	E3 ubiquitin-protein ligase RMA3	IV	At3g47160	RING/U-box superfamily protein	I-IV
At5g02880	E3 ubiquitin-protein ligase UBC4	II	At3g61180	RING/U-box superfamily protein	IV
At2g04240	E3 ubiquitin-protein ligase XERICO	II-IV	At4g33940	RING/U-box superfamily protein	II-III
At5g25350	EIN3-binding F-box protein 2	I-III	At5g01520	RING/U-box superfamily protein	II
At1g23390	F-box protein	IV	At5g03180	RING/U-box superfamily protein	II
At1g26930	F-box protein	III	At5g10650	RING/U-box superfamily protein	II
At1g30200	F-box protein	IV	At5g19430	RING/U-box superfamily protein	II-IV
At1g51550	F-box protein	I-IV	At5g24870	RING/U-box superfamily protein	II
At1g55000	F-box protein	IV	At5g55970	RING/U-box superfamily protein	II-III-IV
At1g70590	F-box protein	II	At4g00335	*RING-H2 FINGER B1A*	IV
At3g12350	F-box protein	II	At5g01880	*RING-H2 FINGER PROTEIN ATL7*	II
At5g27920	F-box protein	III	At2g17450	*RING-H2 FINGER PROTEIN ATL44*	I-IV
At5g43190	F-box protein	III	At2g18670	*RING-H2 FINGER PROTEIN ATL56*	IV
At1g21760	F-box protein 7	II-IV	At5g66160	*RMR1*	IV
At2g42620	F-box protein MAX2	II, IV	At3g60300	RWD domain-containing protein	IV
At4g03030	F-box protein OR23	II-III-IV	At2g22980	*SERINE CARBOXYPEPTIDASE 13*	I
At1g80110	F-box protein PP2-B11	II	At1g01650	*SIGNAL PEPTIDE PEPTIDASE 4*	II-III
At5g57900	F-box protein SKIP1	II-III-IV	At1g45976	*S-RIBONUCLEASE BINDING PROTEIN 1*	II-III-IV
At1g21410	F-box protein SKP2A	II	At3g06380	*TUBBY-LIKE F-BOX PROTEIN 9*	IV
At1g77000	F-box protein SKP2B	II	At1g63800	*UBIQUITIN CONJUGATING ENZYME E2 5 (UBC5)*	I-IV
At4g10925	F-box protein SKIP8	IV	At5g41700	*UBC8*	I
At3g26000	F-box protein SKIP14	II	At4g36410	*UBC17*	I
At4g21510	F-box protein SKIP27	III	At1g64230	*UBC28*	IV
At5g45360	F-box protein SKIP31	IV	At3g17000	*UBC32*	II-III

*The GRN to which each gene belongs to is indicated.*

To test the possibility that these GRNs correspond to a C starvation response, we compared the GRN genes with four lists of genes induced in C-limiting conditions: (i) 26 genes of a robust core of C-signaling response shared by 21 Arabidopsis accessions (Supplementary Dataset S4; Sulpice et al., 2009), (ii) 57 sugar-responsive genes, proposed upstream components of the transcriptional response to sucrose (Supplementary Dataset S4; Osuna et al., 2007), (iii) 429 dark-induced, sugar-repressed genes (Supplementary Dataset S4; Gonzali et al., 2006) and (iv) 507 genes responsive to *AKIN10*, a catalytic subunit of the SUCROSE-NON-FERMENTING-1-RELATED PROTEIN KINASE (SnRK1), which integrates stress and C signals to coordinate energy balance, metabolism and growth (Supplementary Dataset S4; Baena-González et al., 2007).

Genes from these sets appeared in the GRNs at a much higher frequency than expected in a random list (pval 4.5E-11 to 7.5E-215; **Table 2** and Supplementary Figure S4), indicating that the bud dormancy GRNs were very highly enriched in genes typical of a C starvation response.

**Table 2:** C starvation genes are overrepresented in the bud dormancy GRNs.

Geneset	*N*	Freq. (Gen) x100	NExp. (GRN)	NObs. (GRN)	Freq. (GRN) x100	pval	GRN
Core C-signaling	26	0.08	0.230	8	2.7	4.5E-11	GRNI
Sugar-responsive	57	0.17	0.504	18	6.1	1.4E-23	
Dark-induced, sugar-repressed	429	1.28	3.792	95	32.0	7.9E-107	
*AKIN10*-responsive	507	1.51	4.481	56	18.9	2.8E-44	

Core C-signaling	26	0.08	0.219	6	2.1	6.6E-08	GRNII
Sugar-responsive	57	0.17	0.480	10	3.5	4.5E-11	
Dark-induced, sugar-repressed	429	1.28	3.613	72	25.4	2.4E-72	
*AKIN10*-responsive	507	1.51	4.270	63	22.3	1.5E-54	

Core C-signaling	26	0.08	0.210	10	3.7	4.6E-15	GRNIII
Sugar-responsive	57	0.17	0.460	18	6.6	2.7E-24	
Dark-induced, sugar-repressed	429	1.28	3.460	91	33.6	1.8E-104	
*AKIN10*-responsive	507	1.51	4.089	67	24.7	2.6E-61	

Core C-signaling	26	0.08	0.228	19	6.4	2.9E-34	GRNIV
Sugar-responsive	57	0.17	0.500	31	10.5	3.5E-49	
Dark-induced, sugar-repressed	429	1.28	3.766	152	51.5	7.5E-215	
*AKIN10*-responsive	507	1.51	4.451	120	40.7	1.7E-141	

*Four sets of genes induced under conditions of C starvation (Core C-signaling, Sulpice et al., 2009; Sugar-responsive, Osuna et al., 2007; Dark-induced, sugar-repressed, Gonzali et al., 2006; *AKIN10*-responsive, Baena-González et al., 2007) were tested for overrepresentation in the bud dormancy GRNs performing a hypergeometric test. All gene sets were very significantly overrepresented in the GRNs. N(Gen), number of genes of the gene set; Freq.(Gen), frequency of gene sets in the Arabidopsis genome (33602 genes); NExp.(GRN), number of expected genes in the GRN; NObs.(GRN), number of observed genes in the GRN; Freq.(GRN), frequency in the GRN.*

### GSEA Analyses Confirm a C Starvation Response in Dormant Buds

**FIGURE 2:**
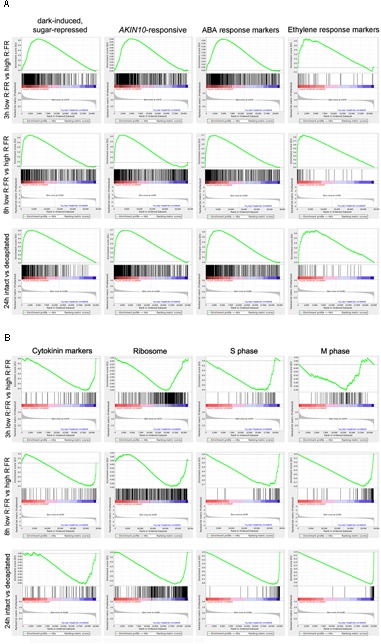
**Gene Set Enrichment Analysis (GSEA) analyses of dormant *vs.* active bud experiments. (A,B)** Enrichment Scores (ES; green line) of selected gene sets that illustrate significant overrepresentation among up- **(A)** or down-regulated genes **(B)**. Barcode-like vertical black lines represent logRatios of genes of each gene set in the ranked ordered data sets. Left (positive logRatios), genes induced in dormant buds; right (negative logRatios), genes repressed in dormant buds.

**FIGURE 3:**
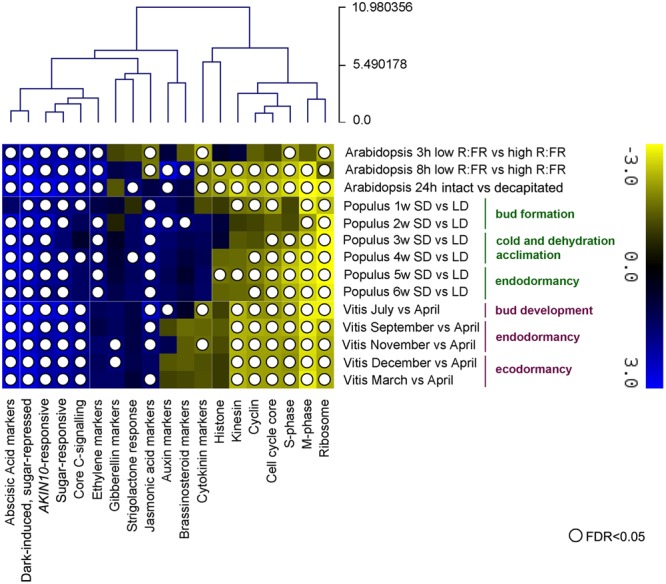
**Summary of GSEA analyses of dormant *vs.* active buds in Arabidopsis, poplar and grapevine.** Clustering of GSEA results for all transcriptomic samples and gene sets based on their normalized enrichment score (NES) for each sample. Complete results are in http://bioinfogp.cnb.csic.es/files/projects/tarancon_et_al_2017_supp/. Positive NES values (blue) are for gene sets overrepresented in the “dormant bud” condition. Negative NES values (yellow) are for gene sets overrepresented in the “active bud” condition. White circles indicate gene sets with a significant statistical overrepresentation (FDR < 0.05). Core C-signaling and Brassinolide (BL) markers in poplar, and ethylene and strigolactone (SL) markers in grapevine contained <10 genes each, which may have prevented obtaining significant results.

We assessed this potential C starvation response by performing a Gene Set Enrichment Analysis (GSEA) using all transcribed genes from each experiment, rather than focusing on the bud dormancy GRNs. GSEA is a statistical approach that allows identification of overrepresented gene sets among differentially up- or downregulated genes of a transcriptomic experiment (Subramanian et al., 2005). For each “active-vs.-dormant bud” experiment, we generated a ranked gene list using relative gene expression levels and False Discovery Rate (FDR) values. We then tested whether gene sets related to a potential C starvation syndrome (sugar-, darkness- and *AKIN10*-responsive genes, ABA, ethylene and CK markers, ribosomal genes, cell cycle and cell division genes; Supplementary Dataset S4) were found toward the top (upregulated) or the bottom (downregulated) of the ranked gene lists. Analyses^1^ confirmed significant overrepresentation of C-signaling, sugar-repressed, *AKIN10*-induced and ABA and ethylene marker genes among those upregulated in the three experiments (**Figures 2A, 3**). In contrast, CK markers, ribosomal genes and S-phase genes were overrepresented among the downregulated genes (**Figures 2B, 3**). Other cell division markers such as M-phase genes, histones and kinesins were overrepresented only in the 8 and 24 h experiments, which suggests they are downregulated at later stages of the process (**Figures 2B, 3**).

All these results suggest that a C starvation syndrome is induced early in the growth-to-dormancy transition in para- and ecodormant axillary buds in Arabidopsis.

### Regulation of the Bud Dormancy GRNs

To find potential master regulators of the C starvation-related bud dormancy GRNs, we searched for overrepresented motifs in the gene promoters (1 kilobase upstream of the transcription start site) of each GRN using Oligo-analysis and Pattern assembly (Rsat; Medina-Rivera et al., 2015). In GRNI and GRNIV, tcTTATCCAc was the most-overrepresented motif (Supplementary Figure S5 and Dataset S5); it contains the sucrose-repressible element TATCCA, bound in rice by the MYB factors OsMYBS1, 2, and 3, which mediate sugar-regulated gene expression (Lu et al., 1998, 2002). We looked for TFs within GRNI and IV that could bind this motif, based on DNA affinity purification sequencing (DAP-Seq) data (O’Malley et al., 2016) or chromatin immunoprecipitation sequencing (ChIP-Seq) data (Song et al., 2016), and that could act as master regulators of the GRNs. The Arabidopsis OsMYBS2 ortholog MYBS2 (At5g08520), which has a role in sugar and ABA signaling (Chen et al., 2016), pertains to GRNI and might bind this motif (Supplementary Figure S6A and Dataset S5). In GRNIV, MYBS2 and three other MYB-related proteins, MYBH/KUA1 (At5g47390), At1g19000 and At1g74840 could bind this motif (Supplementary Figure S6A and Dataset S5). *MYBH/KUA1* has a critical role in dark-induced leaf senescence (Huang et al., 2015). These sugar-regulated genes could be instrumental in coordinating gene expression in GRNI and GRNIV.

The most overrepresented motifs in GRNII and III, gaCACGTGtc, tgaCACGT and gACACGT, overlap with the G-box (CACGTG), which is bound by bZIP, bHLH and NAC proteins (Supplementary Figure S5 and Dataset S5). These motifs are overrepresented in the promoters of C starvation response genes (Cookson et al., 2016). In GRNII, the master regulators of ABA signaling GBF2, GBF3, ABF3 and ABF4, and the senescence-inducing NAC factors NAP, NAC6/ORE1 and ATAF1 as well as NAC047 and NAC3 could bind these motifs (Supplementary Figure S6A and Dataset S5; Guo and Gan, 2006; Balazadeh et al., 2010; Garapati et al., 2015; O’Malley et al., 2016; Song et al., 2016). In GRNIII, NAP, NAC6/ORE1, NAC047, NAC3, NAC102, RD26 (Supplementary Figure S6A and Dataset S5) and possibly NAC19, for which there is no available binding information, might regulate these motifs and promote gene expression.

We confirmed significant enrichment of the GRNs in the target genes of these TFs by using DAP-Seq and ChIP-Seq data (O’Malley et al., 2016; Song et al., 2016); their numbers in the GRNs were significantly higher than expected in a random gene list (pval < 0.01). For instance, the number of gene targets for NAC102, RD26 and ABF4 was 6, 3.2, and 3.4 times higher, respectively; for the remainder, this value was between 1.7 and 2.7 times higher than predicted (Supplementary Figure S6B).

All these results indicate that four interrelated GRNs associated to a C starvation response are induced in para- and ecodormant Arabidopsis buds. MYB-related, bZIP and NAC TFs could have a key role in the regulation of these GRNs. A large proportion of the genes in the GRNs are rapidly repressed by sugar and upregulated by *AKIN10*. They are tightly coregulated with or directly involved in sugar signaling and metabolism, autophagy, senescence, catabolism of lipids and proteins, and ABA and ethylene signaling. This response is also associated with downregulation of CK signaling, protein synthesis and cell division, all conditions that lead to the cell and tissue growth arrest typical of dormant buds.

### Conservation of Bud Dormancy GRNs in Arabidopsis, Poplar and Grapevine

We investigated whether the GRNs related to a C starvation syndrome identified in Arabidopsis were also induced during the growth-to-dormancy transition in buds of the woody plant species, poplar and grapevine. We studied two public transcriptomic experiments in which apical buds of poplar (Ruttink et al., 2007) or axillary buds of grapevine (Díaz-Riquelme et al., 2012) underwent dormancy. To induce dormancy, shoot apices of poplar plants grown in long days (LD, 16 h light-8 h darkness) were exposed to 1–6 weeks of short days (w SD, 8 h light-16 h darkness) (Ruttink et al., 2007). During treatment, the shoot apices developed into buds (1–3 w SD), grew adapted to dehydration and cold (3–6 w SD), and became dormant (5–6 w SD). Samples were collected weekly. Díaz-Riquelme et al. (2012) collected monthly samples of axillary buds of grapevine plants grown in natural conditions in the northern hemisphere. Grapevine axillary buds are formed between April and May; in July and August they grow, undergo flowering and develop inflorescence meristems, enter endodormancy at the end of September, and exit dormancy by the end of November. They remain ecodormant throughout December, until environmental conditions become benign around March, when they sprout (Martìnez de Toda Fernaàndez, 1991; Díaz-Riquelme et al., 2012).

We analyzed the expression patterns of the poplar and grapevine orthologs of the GRNI-IV genes. Of 838 Arabidopsis genes in these GRNs, we identified 390 poplar and 421 grapevine orthologs (Supplementary Dataset S6). In both species, we studied gene expression relative to levels in the “active bud” sample (LD in poplar, April in grapevine). In general, a large proportion of the bud dormancy gene orthologs were significantly induced at most time points in poplar and grapevine buds (Supplementary Figure S7), which supports a conservation, during the growth-to-dormancy transition in these woody species, of the responses found in Arabidopsis. In poplar, the global induction appeared to increase over the weeks in SD, especially for GRNII and III genes. In contrast, grapevine gene induction was detectable throughout the year (Supplementary Figure S7).

**FIGURE 4:**
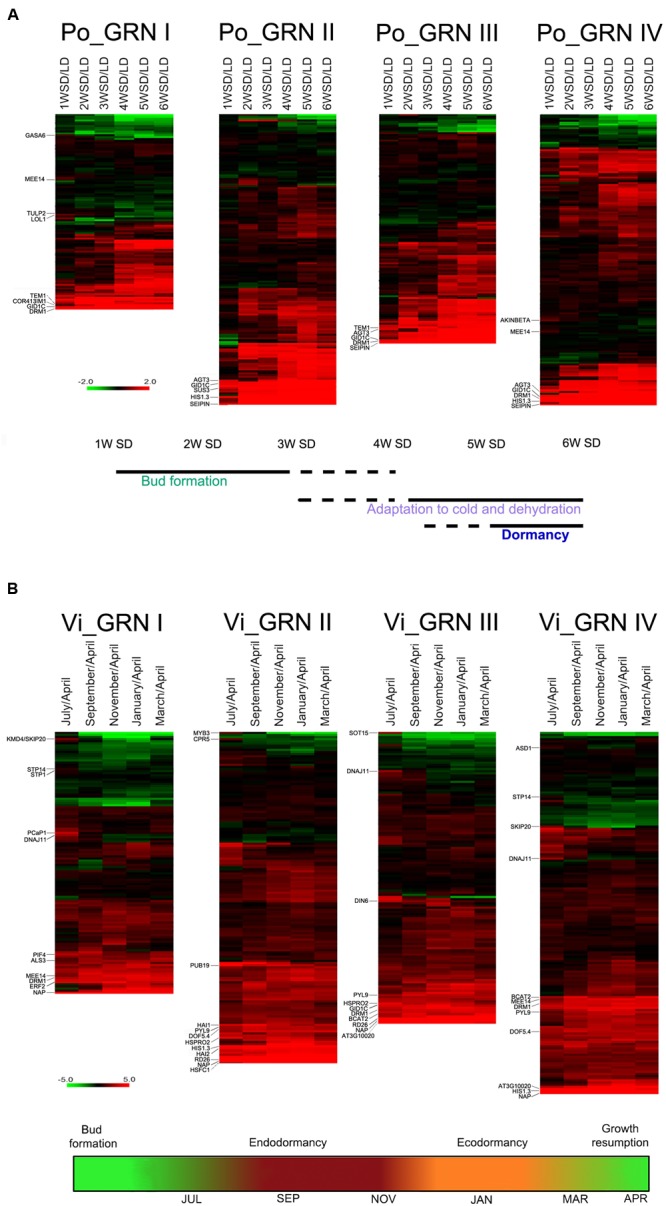
**Expression profiles of bud dormancy genes in poplar and grapevine.** Heatmap of gene expression for poplar (**A**, Po_GRN) and grapevine (**B**, Vi_GRN) orthologs of the Arabidopsis GRN genes. Log2 ratios of normalized gene intensities in each time point vs. normalized gene intensities on the active bud sample are indicated. For poplar and grapevine the “active bud” sample are LD and April, respectively. In red and green, genes up- and downregulated in dormant buds respectively. Genes mentioned in the text are indicated. Schematic representations based on information from Ruttink et al. (2007) **(A)** and Díaz-Riquelme et al. (2012) **(B)**, below indicate the proposed developmental stage of buds in each time point.

A group of genes showed high expression levels from the earliest stages (1–3 w SD in poplar and July in grapevine), weeks/months before endodormancy onset, and throughout the experiment (**Figure 4** and Supplementary Dataset S6). In poplar, these were *DRM1, HIS1-3, GID1C, COR413IM1, ALANINE:GLYOXYLATE AMINOTRANSFERASE 3 (AGT3), SUCROSE SYNTHASE3* (*SUS3*), *TEMPRANILLO1 (TEM1)* and *SEIPIN* (**Figure 4A**). In grapevine, they were *DRM1, HIS1-3, DIN6, PIF4, CBP1/MEE14, HSPRO2, EXL4, BCAT2* and *ATY13/MYB31, ERF2*, ABA receptor *PYL9*/*ABI1*, the protein phosphatases 2C *HAI1/SAG113* and *AIP1/HAI2*, involved in ABA signaling and sucrose sensitivity (Lim et al., 2012), *DOF5.4, HSFC1, PLANT U-BOX 19, GOLS1*, senescence factor *NAP, RD26, ALUMINUM SENSITIVE 3* (*ALS3*), and oxidative stress-related *At3g10020* (**Figure 4B**).

Other bud dormancy genes were induced exclusively between 1 and 3 w SD in poplar, and in July in grapevine. Early poplar genes were *GASA6*, the sugar-responsive gene *CBP1/MEE14* (Bi et al., 2005), *AKINBETA1* and *LSD ONE LIKE* 1 (*LOL1*), a positive regulator of cell death (Epple et al., 2003) (**Figure 4A** and Supplementary Dataset S6). July-induced grapevine genes were sugar transporters *STP14* and *STP1, KISS ME DEADLY 4/SKP20*, which encodes an F-box protein that negatively regulates the CK response, autophagy factor *ATG8I*, chaperone *DNAJ11, PLASMA-MEMBRANE ASSOCIATED CATION-BINDING PROTEIN 1* (*PCaP1*), *ASD1*, involved in cell wall remodeling (Chávez Montes et al., 2008), and ABA-responsive *MYB3* (**Figure 4B** and Supplementary Dataset S6).

In summary, a large proportion of the genes orthologous to Arabidopsis bud dormancy genes are also induced, either early and transiently or early and constantly during the growth-to-dormancy transition in poplar and grapevine, which supports their functional conservation in these woody species.

### The C Starvation Response Is Conserved in Poplar and Grapevine Buds

To obtain a general view of the transcriptomic responses in these experiments, we performed GSEA similar to that for Arabidopsis, using all genes with proposed Arabidopsis orthologs (8023 genes in poplar and 8390 in grapevine) (Ruttink et al., 2007; Díaz-Riquelme et al., 2012).

The sugar- and *AKIN10*-responsive gene sets were overrepresented among upregulated genes from 1 w SD in poplar, and July in grapevine, and were also induced throughout the treatment/year (**Figure 3**). This finding confirms that the C starvation response begins early, long before endodormancy onset, and underlies the entire process. The ribosomal gene set was constitutively overrepresented among downregulated genes in all three species, which confirmed that inhibition of protein synthesis is an early and sustained response in buds entering dormancy. General downregulation of cell cycle and cell division genes was also observed in grapevine, whereas in poplar, cell division gene sets were repressed more gradually and reached maximum repression at 5 w SD. In contrast to Arabidopsis, histones were not significantly downregulated in the woody species. Nevertheless, C starvation response gene sets (upregulated) and cell growth-related gene sets (downregulated) clustered together in the three species.

Unlike the gene sets discussed above, hormone responses did not appear to be strongly conserved among species, suggesting more relaxed evolution of these pathways. Whereas ABA-related genes were induced constitutively in grapevine, ABA and ethylene responses were induced from 2 w SD onward in poplar, in accordance with previous observations (Ruttink et al., 2007). CK signaling is repressed in Arabidopsis, but not notably in poplar or grapevine. An early, extended response to the senescence-associated hormone jasmonate (MJ) was repressed in two Arabidopsis experiments, and was induced in most poplar and grapevine samples (**Figure 3**).

This results indicate that an early and sustained sugar-starvation response associated with downregulation of ribosomal and cell cycle proteins is conserved in buds of Arabidopsis, poplar and grapevine, and might constitute a core response of buds entering dormancy in the angiosperms.

### Cell Type-Specific Gene Expression of Bud Dormancy Genes in the Shoot Apex

To further analyze the function of the genes induced during the C starvation response in buds, we selected those most highly expressed in Arabidopsis, poplar and grapevine, to determine the cell types in which they are expressed. We used a high-resolution gene expression database of the Arabidopsis shoot apex, which contains the same tissues as axillary buds: meristem and leaf primordia. This database comprises gene expression profiles of different cell populations obtained by fluorescence-activated cell sorting (L1, L2 and L3 layers, central (CZ) and peripheral zone (PZ), leaf primordia, xylem and phloem) (Yadav et al., 2014). As we cannot rule out that the expression levels of these genes change in dormant axillary buds, we used this database for qualitative rather than quantitative analysis, to identify the cell types in which these genes were expressed most abundantly.

**FIGURE 5:**
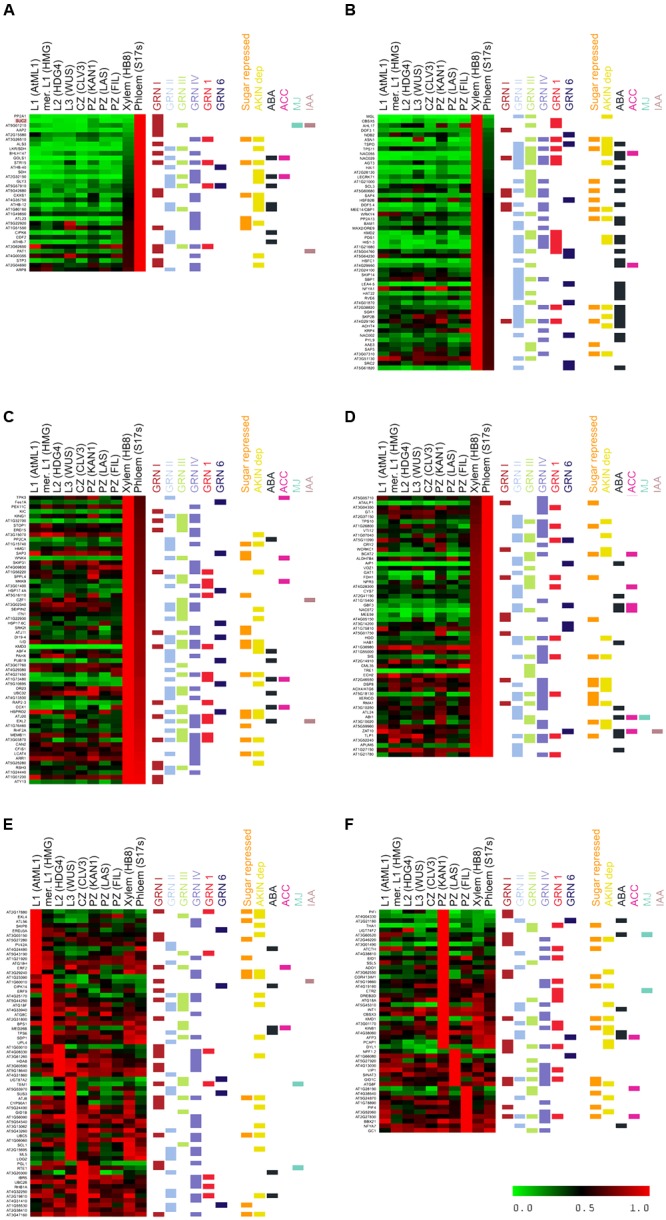
**Cell type-specific expression of bud dormancy genes in the Arabidopsis shoot apical meristem (SAM).** Heatmap of bud dormancy gene expression in SAM cell types, normalized for each gene relative to the cell type with the highest expression levels (1.0, red). No expression (0.0), green. Different cell types are indicated on top. Horizontal color lines on the right indicate gene sets to which each gene belongs. **(A)** Genes expressed almost exclusively in phloem. **(B)** Genes expressed almost exclusively in xylem. **(C,D)** Genes expressed preferentially in phloem and xylem. **(E)** Genes expressed preferentially in layers 1, 2, 3 (L1, L2, L3), meristem (mer) and central zone (CZ). **(F)** Genes expressed preferentially in peripheral zone (PZ).

Many of the most highly induced genes in buds were expressed preferentially in the vasculature (**Figures 5A–D**); sugar-related genes *SUC2, STP3, TPS11, GOLS1*, and TF *HB-40, HB-7, HB-12, PAT1* and *CDF2* were expressed exclusively in phloem (**Figure 5A**). Many *ABA*-related genes (*PYL9, HAI1, NAP, NAC055, NAC002, NFYA1, HAT22, RVE6, LEA4-5, DOF5.4, HIS1-3* and *TSPO*), *SCR*, the F box-encoding genes *MAX2* and *KMD2, CBP1/MEE14, AGT3*, and *CBSX5* were expressed almost exclusively in xylem (**Figure 5B**). Other *ABA-*related genes (*ABF4, HAB1, HAI2, GBF3, RD26, MYB31, RAP2.3*) as well as *KING1, TPS10, TRE, XERICO, EXL2, KMD3, VOZ1, SIS* and *PUB19* were expressed in both xylem and phloem (**Figures 5C,D**). In the *CLV3/WUS* expression domain, we found *TEM1*, protein kinase *CIPK14* that interacts with SnRK1 (Yan et al., 2014), *SUS3, UDP-GLUCOSYL TRANSFERASE 87A2* (*UGT87A2*) and *6 PHOSPHOGLUCONOLACTONASE 1 (PGL1*) (**Figure 5E**). The genes *PIF1, PIF4, EXL4, ADAGIO (ADO1), ETR2* and *COR413 IM1* accumulated preferentially in leaf primordia (**Figure 5F**). Other strongly expressed genes such as *DRM1, PCAP1, KMD1*, and *AFP3* were found in both vascular tissue and leaf primordia, and *GID1C* in xylem and the peripheral zone of the meristem. The autophagy genes *ATG8F, ATG8C, ATG18A, ATG18F, ATG18H*, the senescence gene *SAP3*, as well as *ERF2, GID1B, BYPASS* and *HISTONE DEACETYLASE 8* were widely expressed throughout the meristem.

In summary, whereas ABA signaling occurs mostly in the xylem, sugar signaling in the phloem, and ethylene in the meristem proper (Yadav et al., 2014), autophagy and arrest of cell growth take place throughout the meristem. This suggests that cell-to-cell communication and movement of signaling molecules, hormones and proteins must take place across different cell types in buds entering dormancy.

### Bud Dormancy Early Markers

It is of great interest to identify robust, universal markers that allow diagnosis of axillary bud status. These markers should be induced early, and their expression be sustained in para-, eco-, and endodormant buds throughout angiosperms. Based on our analysis, several genes met these criteria. We tested further four of them: *DRM1, HIS1-3, GID1C*, and *NAP*. *DRM1* and *HIS1-3* were expressed at high levels in the Arabidopsis, poplar and grapevine experiments. *DRM1* is a well-known dormancy marker in herbaceous and woody plants species (Stafstrom et al., 1998; Park and Han, 2003; Tatematsu et al., 2005; Aguilar-Martínez et al., 2007; Kebrom et al., 2010; Wood et al., 2013). It is repressed by sugar, which supports its strong association to low sugar levels and dormancy. ABA-responsive *HIS1-3* is upregulated before ABA signaling in poplar and grapevine buds. *GID1C*, which encodes a gibberellin receptor, is expressed at high levels in the three Arabidopsis experiments and in poplar (**Figure 4A**). The senescence-promoting gene *NAP* is expressed at very high levels in Arabidopsis and throughout the year in grapevine (**Figure 4B**). Both *GID1C* and NAP belong to the four bud dormancy GRNs (Supplementary Dataset S1).

**FIGURE 6:**
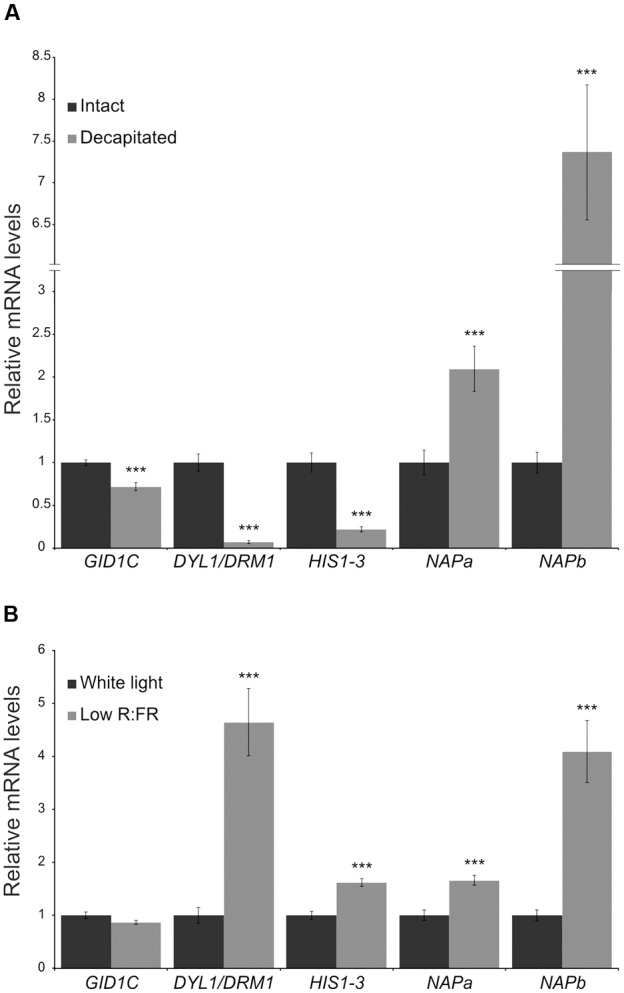
**Expression levels of candidate bud dormancy markers in potato.** Transcript levels of *DRM1, HIS1-3, GID1C* and *NAP* potato ortholog genes in aerial axillary buds were analyzed by quantitative PCR. **(A)** Intact and decapitated plants, 10 h after treatment. **(B)** Plants exposed for 10 h to white light or to a low red:far-red light ratio. Values are mean ± SEM (N_A_ = 5, N_B_ = 4). Each biological replicate is a pool of 16 axillary buds. ^∗∗^pval < 0.01; ^∗∗∗^pval < 0.001; two-tailed Student’s *t*-test.

We tested whether expression of these genes also correlated with bud dormancy in axillary buds of potato (*Solanum tuberosum*, Solanaceae, Asteridae), a species only distantly related to Arabidopsis, poplar (both Rosidae) and grapevine (basal angiosperms). We identified the potato ortholog genes for the four candidates. In the case of *NAP*, we found two potato paralogs (*NAPa* and *NAPb*). We studied their mRNA levels in buds of plants treated for 10 h with white light (W) or with W supplemented with far red light (W+FR), a treatment that promotes axillary bud dormancy in potato. We also compared mRNA levels in buds of intact and decapitated plants. Whereas *DRM1* and *HIS1-3* were confirmed as reliable markers of bud dormancy in potato, *GID1C, NAPa* and *NAPb* did not respond as anticipated in decapitated plants; *GID1C* was not upregulated in low R:FR and *NAPa/NAPb* were highly induced after decapitation (**Figure 6**).

## Discussion

### Carbon Availability, a Key Signal for Growth

In all eukaryotes, cell proliferation and growth demand high carbohydrate levels for energy generation and macromolecule synthesis. Correspondingly, low C availability promotes a reduction in growth rate in order to retain sufficient C to support essential maintenance functions (Rolland et al., 2006). In Arabidopsis, sugar availability has a great influence on growth and development, both in seedlings and adult plants. C-limiting conditions (e.g., sucrose depletion, night extensions, short-day photoperiods, starchless mutants) trigger a suite of transcriptional responses that lead to growth cessation, and which include repression of genes involved in anabolism, protein synthesis, cell division, cell cycle, and DNA synthesis and repair (Moore et al., 2003; Smith and Stitt, 2007; Wiese et al., 2007).

Regarding shoot branching, it has been shown that sugar availability to buds plays a major role in its control in pea and rose (Mason et al., 2014; Barbier F. et al., 2015). In agreement, we have found that induction of GRNs typical of tissues undergoing C starvation precedes and underlies the bud growth-to-dormancy transition in Arabidopsis, poplar and grapevine. This is concomitant with transcriptional repression of ribosomal and cell-cycle genes, responses typical of buds entering dormancy as well as of tissues undergoing C limitations (e.g., Thimm et al., 2004; Smith and Stitt, 2007; González-Grandío et al., 2013). Indeed it is possible that bud dormancy is a manifestation and a consequence of the observed C starvation syndrome.

### Dormancy-Promoting Stimuli and the C Starvation Response

How is this C starvation response induced? In apical dominance it has been proposed that the growing shoot apex acting as a sugar sink might limit sugar availability to axillary buds so this can be the direct trigger of the response (Mason et al., 2014). Tre6P, a metabolite that acts as a proxy for C status, may also promote signaling in addition to, or instead of direct sugar sensing (Paul et al., 2008; Lunn et al., 2014). This would be in agreement with the observation that plants that express microbial trehalose-phosphate synthase (*TPS)* genes show increased shoot branching (Goddijn et al., 1997) and maize plants with a mutation in the trehalose-phosphate phosphatase gene *RAMOSA3* have altered inflorescence branching (Satoh-Nagasawa et al., 2006).

Nevertheless, it is likely that the syndrome is not only induced by an actual sugar shortfall, but also by cues that inform of current or future suboptimal conditions which may affect energy availability and/or interfere with respiration and C assimilation (Baena-González et al., 2007; Baena-González and Sheen, 2008). Seasonal environmental changes that perturb these processes (e.g., daylength shortening, light levels, temperature, water availability) may trigger acclimatory signaling pathways that anticipate C limitations (Smith and Stitt, 2007). Those and other stimuli could feed into regulatory networks that economize resources locally, to result in a moderation of growth rate in axillary meristems and buds.

In two of the Arabidopsis experiments examined, the dormancy-inducing stimuli was an exposure to low R:FR light ratio. Low R:FR light is interpreted by plants as a situation with limited light available for photosynthesis. It severely reduces the expression of photosynthesis-related genes (Cagnola et al., 2012) and induces cell-wall remodeling in stem and petioles, which may divert carbohydrates away from axillary buds (Sasidharan et al., 2010). Furthermore, low R:FR promotes ethylene and ABA signaling and CK degradation (Carabelli et al., 2007; Cagnola et al., 2012), hormonal responses tightly linked to the C starvation response (see below).

In the poplar and grapevine studies, the sugar-repressed networks are induced in buds soon after beginning of daylength shortening: in poplar at 1 w SD; in grapevine, in July, when daylength shortening has just begun (June 21), even though buds are still growing. Short-day photoperiod leads to localized flower and seed abortion associated with low levels of C in Arabidopsis (Lauxmann et al., 2016). Likewise, in poplar a measurable shortage of sugar availability is detectable after 1 w SD (Ruttink et al., 2007). Under the natural conditions in which grapevine plants are grown, daylength shortening and C limitations are progressive, but relatively small changes in C balance may trigger the response. Indeed in Arabidopsis even minor alterations in C status, well before C starvation, lead to notable changes in C-related signaling and response (Usadel et al., 2008). In addition, genetic pathways that sense photoperiod might help anticipate and adapt to impending C-limiting conditions in short days. These pathways, controlled by phytochromes, circadian clock, and genes controlling flowering time (Horvath, 2009), may regulate and establish crosstalk with the C starvation response. Indeed, sugars affect the expression of clock genes (Haydon et al., 2013) and conversely, the clock regulates carbohydrate metabolism (Smith and Stitt, 2007). Phytochromes, which monitor changes in R:FR and in day-length, also regulate SD-induced endodormancy in woody species (Johnson et al., 1994; Reed et al., 1994; Olsen et al., 1997; Neff and Chory, 1998; Monte et al., 2003; Ruonala et al., 2008; Franklin and Quail, 2010). Changes in low R:FR light ratio or photoperiod might therefore trigger partially overlapping responses, including potential anticipation of a C-limiting situation.

Although it has not been analyzed in this work, coordination between C and N metabolic pathways probably affect this process as well, as sugar responses depend significantly on the N status of the plant.

### The C Starvation Syndrome in Axillary Buds: Sugar Signaling

**FIGURE 7:**
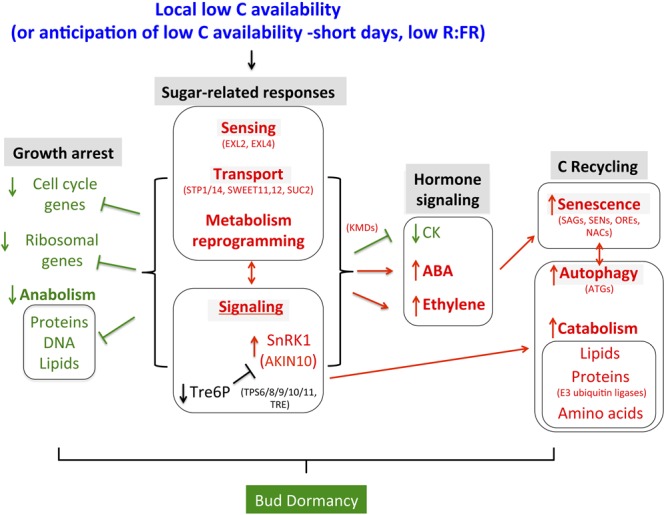
**Summary of responses observed in buds entering dormancy in the context of a potential C starvation response.** Relationships are based on data obtained in Arabidopsis (see Discussion). Some relevant Bud dormancy genes are indicated.

The C starvation syndrome comprises a cascade of transcriptomic events that culminate in changes in growth and C balance (**Figure 7**). These events include induction of genes involved in transcriptional regulation, sugar sensing, transport and signaling, catabolism (i.e., amino acid and lipid degradation), protein ubiquitination and degradation, hormone signaling, autophagy and senescence. Genes required for growth, such as ribosomal, cell cycle and anabolism-related genes become downregulated (Thimm et al., 2004). Buds entering dormancy in Arabidopsis, poplar and grapevine show induction of genes of the former categories and repression of genes of the latter categories.

*EXORDIUM-like (EXL*)*2* and *EXL4* are bud dormancy genes potentially involved in sugar sensing. They are induced in extended night treatments in Arabidopsis seedlings, in accordance with a role under C-limiting conditions (Schröder et al., 2012). Their close paralogs, *EXORDIUM* (*EXO*) and *EXL1*, are proposed to integrate apoplastic C status with intracellular responses (*EXO*) (Lisso et al., 2013) and to control primary and long-term adaptation to C starvation (*EXL1*) (Schröder et al., 2011, 2012).

Several sugar transporters are also induced in dormant buds. These are *STP1*, one of the most rapidly and prominently downregulated genes in response to sugars (Price et al., 2004; Cordoba et al., 2015), *STP14*, which is strongly repressed by sugars (Büttner, 2010) and the sucrose efflux transporters *SWEET11* and *SWEET12*, which act with the sucrose/proton symporter *SUT1/SUC2* for phloem loading and long-distance transport (Chen et al., 2012).

Sugar signaling involves Tre6P (Paul et al., 2008; Lunn et al., 2014) and four class-II *TPS, TPS8, TPS9, TPS10*, and *TPS11*, are induced in buds entering dormancy. They belong to a core C-signaling response, are usually strongly upregulated in C starvation, and are *AKIN10*-responsive (Contento et al., 2004; Price et al., 2004; Thimm et al., 2004; Baena-González et al., 2007). Although their proteins may be catalytically inactive, they might modulate other TPSs or act as Tre6-P sensors (Lunn, 2007). In addition, the SnRK1 protein-kinase, a central regulator of growth in response to C availability (Baena-González et al., 2007), is likely to have a key role in the induction of bud dormancy (see below).

### Ethylene, ABA, CK, Senescence and Autophagy during Bud Dormancy

Ethylene and ABA signaling are induced during the bud dormancy transition in Arabidopsis, which agrees with studies that associated these hormones with bud dormancy in many other herbaceous and woody species (Suttle, 1998; Ruonala et al., 2006; Rohde and Bhalerao, 2007; Ruttink et al., 2007; Horvath et al., 2008; Díaz-Riquelme et al., 2012; González-Grandío et al., 2013, 2017; Reddy et al., 2013; González-Grandío and Cubas, 2014; Yao and Finlayson, 2015). We propose that ethylene and ABA responses are closely connected to the C starvation syndrome. Indeed, many mutants with altered responses to sugars have impaired ethylene or ABA signaling (Zhou et al., 1998; Arenas-Huertero et al., 2000; Finkelstein and Lynch, 2000; Huijser et al., 2000; Laby et al., 2000; Rook et al., 2001; Cheng et al., 2002; Yuan and Wysocka-Diller, 2006), and there is compelling evidence of crosstalk between sugar sensing and ethylene and ABA response. C starvation leads to induction of ethylene and ABA-related genes, whereas sugar treatment has the opposite effect (Laby et al., 2000; Rook et al., 2001; Brocard et al., 2002; León and Sheen, 2003; Yanagisawa et al., 2003; Thimm et al., 2004; Buchanan-Wollaston et al., 2005; Rolland et al., 2006). Thus C starvation signaling could trigger ethylene and ABA responses in buds. Indeed, the reduction in sugar levels detected in poplar buds during the 1 w SD is suggested to induce ethylene signaling, followed by ABA signaling (Ruttink et al., 2007).

One role of ABA and ethylene in the C starvation syndrome is induction of senescence, a genetically programmed process that promotes degradation of cell components and macromolecules, remobilizes nutrients, and optimizes resources to supply energy and C skeletons. Ethylene and ABA activate senescence-related genes and senescence induces ABA signaling (Abeles et al., 1988; Zeevaart and Creelman, 1988; Zacarias and Reid, 1990; Reid and Wu, 1992; Weaver et al., 1998; Seo et al., 2000; Yang et al., 2003; Buchanan-Wollaston et al., 2005; Lim et al., 2007). It is noteworthy that the potential master regulators of GRNII and III, *ATAF1, ORE1/NAC6* and *NAP*, are ABA-induced factors that control senescence. *ATAF1* induces a C starvation transcriptome and ABA biosynthesis (Jensen et al., 2013; Garapati et al., 2015). *NAP* activates *SAG113/HAI1* and controls expression of *ABSCISIC ALDEHYDE OXIDASE3* (*AAO3*), encoding an enzyme that catalyzes the final steps of ABA synthesis (Guo and Gan, 2006; Yang et al., 2014). *ORE1* controls the expression of at least 78 SAGs and might also promote DNA degradation (Balazadeh et al., 2010; Matallana-Ramirez et al., 2013; Kim et al., 2014). *HAT22, GBF2, GBF3, ABF3* and *ABF4* are additional bud dormancy genes related both to senescence and ABA (Lin and Wu, 2004; Rivero et al., 2007; Song et al., 2016). Remarkably, *MAX2/ORE9*, which encodes an F-box involved directly in strigolactone perception and signaling and has a critical role in the control of shoot branching, also promotes senescence (Woo et al., 2001; Stirnberg et al., 2002). Finally, the MYB genes *At1g19000* and *At1g74840*, proposed to be master regulators of GRNI and IV, are responsive to dark-induced senescence (Lin and Wu, 2004).

In contrast, CK signaling is antagonistic to senescence, and a reduction in CK levels is a key signal for senescence initiation in Arabidopsis (Gan and Amasino, 1995; Kim et al., 2006). Consistent with this, in Arabidopsis dormant buds CK signaling is reduced, and in other species CK levels have also been reported to be reduced relative to active buds (Turnbull et al., 1997; Dun et al., 2012; Roman et al., 2016). Consistently, four genes encoding F-box proteins that promote the ubiquitination and degradation of ARR factors [*KISS ME DEADLY* (*KMD*)1-4] are bud dormancy genes.

Autophagy is another process induced by C starvation (Izumi et al., 2013) and whose markers (*ATG* genes) are upregulated in buds entering dormancy. This is a process by which cytoplasmic components and organelles are transported to the vacuole, where they are broken down and recycled. Under C-limiting conditions it contributes to plant energy availability (Aubert et al., 1996; Rose et al., 2006; Izumi et al., 2013). Autophagy is associated with induction of lipid degradation and upregulation of E2- and E3-ubiquitin ligase components, which promote proteasomal-dependent protein degradation (Thompson and Vierstra, 2005). We have found a remarkable number of bud dormancy genes related to autophagy, ubiquitination, protein degradation and lipid catabolism, many of them controlled by SnRK1 (see below).

### SnRK1 Could Have a Pivotal Role during the Bud Growth-to-Dormancy Transition

SnRK1, a protein-kinase active in low energy conditions, promotes catabolism and represses anabolism, cell division and growth. Our transcriptomic data indicates that it may play an important role during the bud growth-to-dormancy transition. SnRK1 affects expression of robust dormancy markers such *HIS1.3* and *DRM1*, and the potential master regulator of GRNI and GRNIV, *MYBH/KUA1*. In buds entering dormancy, the SnRK1 β subunit *AKINBETA1*, whose mRNA levels correlate directly with night duration (Pokhilko et al., 2014), is induced. Most importantly, our GSEA analysis indicates that the transcriptional network downstream of the catalytic SnRK1 α subunit, *AKIN10*, is significantly induced from the earliest stages of growth-to-dormancy transition in Arabidopsis, poplar and grapevine buds, and is maintained in para-, eco- and endodormant buds. Many of the abovementioned genes involved in sugar sensing, signaling, autophagy and repression of CK signaling are *AKIN10*-dependent, including *EXL4, STP1/14, SWEET11/12, TPS8/9/19/11, AKINBETA, ATG8E/F/G/H, ATG18F/G*, and F-box genes *KMD1, 3, 4*. SnRK1 also causes downregulation of a large number of ribosomal genes, another conserved significant effect detected by our GSEA analysis. SnRK1 could also be responsible for at least part of the observed induction of the ubiquitination machinery and lipid degradation.

### A Conserved Core C Starvation Response Underlies Bud Dormancy in Angiosperms

Bud dormancy is an adaptive response present in all angiosperms. It prevents shoot development when endogenous or environmental conditions are unfavorable for sustained growth. It has great impact on reproductive success, productivity and survival, and must have been influential in the colonization of habitats with fluctuating conditions.

We have found induction of a conserved C starvation syndrome that precedes and underlies the growth-to-dormancy transition in buds of three distantly-related species, one herbaceous (Arabidopsis) and two woody (poplar and grapevine). This transcriptional response, composed by several interconnected GRNs, comprises ortholog genes in Arabidopsis, poplar and grapevine, as gene sets generated in Arabidopsis were used to detect the response in the woody species. Furthermore, this syndrome has been observed is several unrelated experiments, regardless the stimulus that promoted dormancy, either environmental (low R:FR, short-day photoperiods) or endogenous (apical dominance). This remarkable conservation suggests that a syndrome aimed at adapting to C-limiting situations is deeply rooted in the control of shoot meristem and bud development across angiosperms. Bud dormancy might thus be an ancestral response directly resulting from this C starvation syndrome, coordinated by different pathways that sense and/or anticipate situations on low C availability and feed into this core response to prevent untimely growth and development.

## Materials and Methods

### Identification of Coregulated Genes in Bud Dormancy GRNs

Bud dormancy genes (Supplementary Figure S1) were obtained from González-Grandío and Cubas (2014). Coregulation of the 78 bud dormancy genes was analyzed by hierarchical clustering (Hcluster, ATTED-II, Obayashi et al., 2007). Additional coregulated genes were obtained using CoEx-Search (ATTED-II, Obayashi et al., 2007). The 300 genes most coregulated with each cluster were selected. These genes were validated for induction in dormant buds in the original arrays (Tatematsu et al., 2005; González-Grandío et al., 2013; Reddy et al., 2013). Only genes upregulated (positive fold change FC ≥ 1.2) in at least one experiment in dormant buds were included in the lists of bud dormancy GRNs (Supplementary Figure S8).

### Functional Annotation of Bud Dormancy GRNs

Automated function prediction for the GRNs was carried out using GO analyses. The PANTHER classification system (Mi et al., 2017) was used to identify overrepresented biological process ontologies using a statistical overrepresentation test followed by Bonferroni correction for multiple testing. TAIR10 version of *Arabidopsis thaliana* genome was used as reference. We selected ontologies with a pval < 0.05. In addition, Mapman bins (Thimm et al., 2004) were added to all the genes in Supplementary Dataset S1.

### Gene Set Enrichment Analysis

Gene Set Enrichment Analysis (Subramanian et al., 2005) was used to identify gene sets whose genes are overrepresented in different conditions. The GSEA method evaluates whether these genes occur preferentially toward the top or bottom of a ranked list. Enrichment scores are calculated using “weighted” statistics. For each sample, we calculated the log2 ratios of normalized gene intensities vs. normalized gene intensities of the “active bud” sample: white light-treated buds for González-Grandío et al. (2013); High R:FR-treated n-2 buds for Reddy et al. (2013); Buds of decapitated plants for Tatematsu et al. (2005); Buds of LD-grown poplars for Ruttink et al. (2007), April grapevine buds for Díaz-Riquelme et al. (2012). Genes were ranked by their log2 ratios calculated as the difference between normalized log intensity in the “dormant bud” condition minus normalized log2 intensity in the “active bud” condition. Intensity expression values were obtained from the references above. Gene sets for hormone markers were obtained from Nemhauser et al. (2006) and Mashiguchi et al. (2009). Gene sets related to sugar and *AKIN10* responses were obtained from Sulpice et al. (2009) (Core C-signaling), Osuna et al. (2007) (Sugar-responsive), Gonzali et al. (2006) (Dark-induced, sugar-repressed), and Baena-González et al. (2007) (*AKIN10*-responsive). The other gene sets are from González-Grandío et al. (2013). The GSEA Normalized Enrichment Score for all gene sets in all comparisons were clustered with TM4 Multi Experiment Viewer (MeV, Saeed et al., 2003). Tree was generated by the hierarchical clustering method (HCL) using Euclidean distance and average linkage options. Complete results are in http://bioinfogp.cnb.csic.es/files/projects/tarancon_et_al_2017_supp/.

### Promoter Motif Analysis

Sequences (1 kb) 5′ of the transcription start site of the bud dormancy GRN genes were retrieved with Sequence Bulk Download^2^. Overrepresented 6-8mer motifs were identified with Motif discovery (RSAT, Medina-Rivera et al., 2015). The oligo-analysis tool was used to find significantly overrepresented motifs, which were assembled into frequency matrices with pattern-assembly and default parameters. Matrices were converted into consensus motifs with convert-matrix and represented using WebLogo (Crooks et al., 2004).

### Generation and Visualization of Poplar and Grapevine Expression Datasets

For each time point we calculated the log2 ratios of normalized gene intensities vs. normalized gene intensities on LD (active buds). Expression data was visualized and clustered with MeV. Tree was generated by HCL, using Euclidean distance and average linkage options.

### Cell-Type Specific Shoot Apex Expression of Bud Dormancy GRN Genes

For each sample, we calculated the log2 ratios of normalized gene intensities vs. normalized gene intensities of the “active bud” sample: LD for poplar, April for grapevine. Expression data for selected bud dormancy genes obtained from Yadav et al. (2014) was visualized and clustered with MeV. Trees were generated by HCL using Euclidean distance and average linkage options.

### Identification of *Solanum tuberosum* Orthologs

The putative orthologs of Arabidopsis genes were identified by a tblastn search with protein sequences as query in the Spud DB Potato Genomics Resource website^3^. cDNAs showing a high similarity *e*-value with the query were selected. Proteins were aligned with those of Arabidopsis and phylogenetic trees (BioNeighbor joining method, 500 replicates; Gascuel, 1997) were built to identify the most likely orthologs, which were selected for expression studies (Supplementary Figure S9).

### Quantitative-PCR Expression Analyses in *Solanum tuberosum*

Plant growth conditions, experimental design, light treatments, techniques and expression level normalization were as described in Nicolas et al. (2015). For each biological replicate, 8 axillary buds from node 2, and 8 from node 3 were dissected (node 1 = lowest plant node); 4–5 biological replicates were collected for each condition. Primers used are listed in Suplemmentary Table S1.

## Author Contributions

CT, EG-G, JO, and MN, performed experiments. PC, performed experiments, and wrote the manuscript.

## Conflict of Interest Statement

The authors declare that the research was conducted in the absence of any commercial or financial relationships that could be construed as a potential conflict of interest.

## Footnotes

1 http://bioinfogp.cnb.csic.es/files/projects/tarancon_et_al_2017_supp/

2 www.arabidopsis.org

3 solanaceae.plantbiology.msu.edu/index.shtml
