# Supplementary material for: A Conserved Carbon Starvation Response Underlies Bud Dormancy in Woody and Herbaceous Species
Source: Front Plant Sci. 2017 May 23;8:788. doi: 10.3389/fpls.2017.00788 (PMC5440562; doi:10.3389/fpls.2017.00788)
Supplement: Supplementary file 1 [file Presentation_1.PDF]

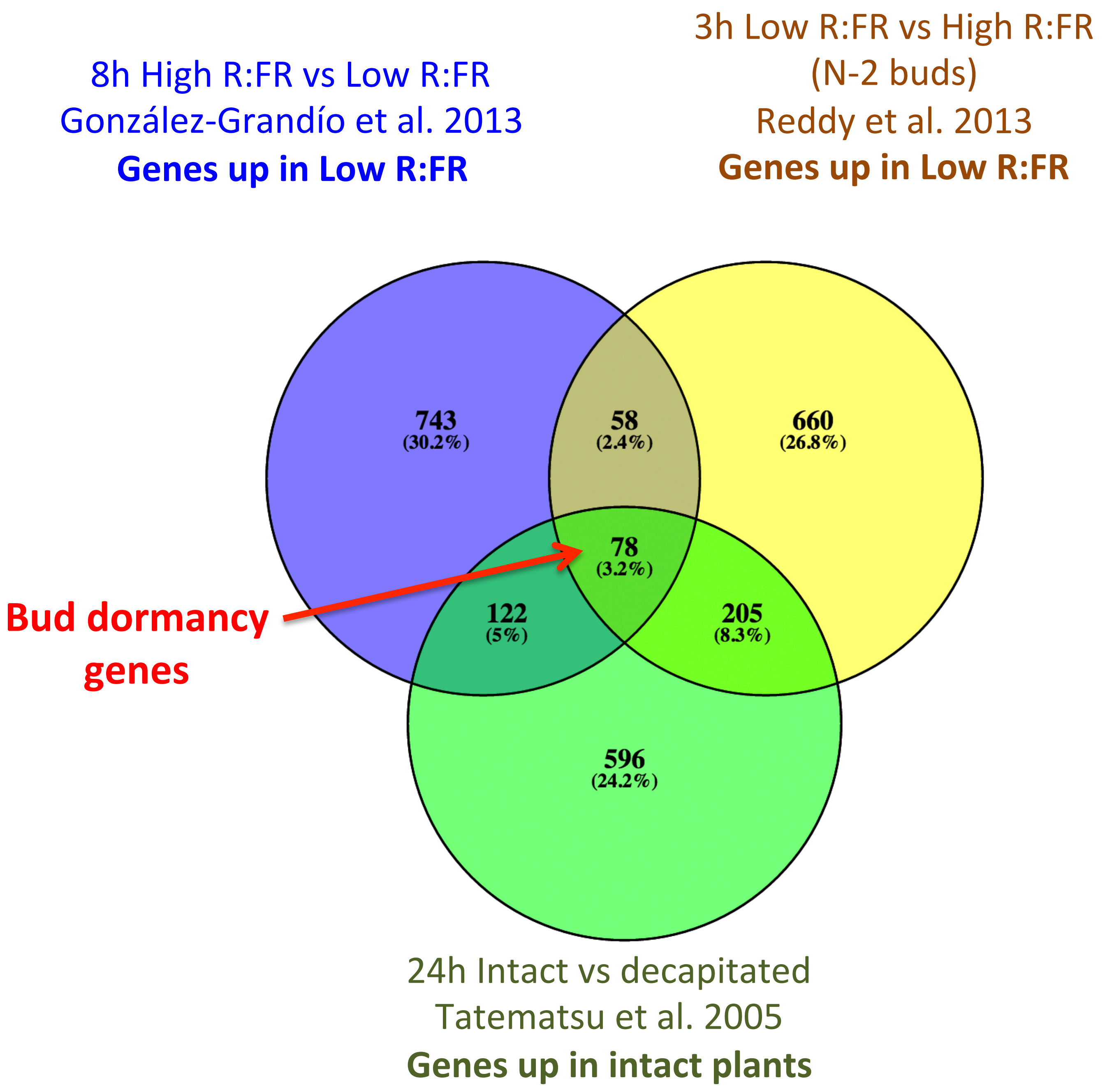

Figure S1. Bud dormancy genes. The 1000 genes most significantly upregulated in dormant buds in the experiments indicated were compared. 78 genes were common to the three gene sets, and were termed Bud dormancy genes (González-Grandío and Cubas 2014).

A

Number of Genes	GRN I (N = 297)		GRN II (N = 283)		GRN III (N = 271)		GRN IV (N = 295)	
Experiment	N up	N down	N up	N down	N up	N down	N up	N down
3h Low R:FR vs High R:FR	15	169	6	242	9	226	0	284
8h High R:FR vs Low R:FR	176	13	167	2	166	5	144	1
24h Intact vs decapitated	0	289	1	264	4	248	0	284
	% up	% down	% up	% down	% up	% down	% up	% down
3h Low R:FR vs High R:FR	5.1	56.9	2.1	85.5	3.3	83.4	0.0	96.3
8h High R:FR vs Low R:FR	59.3	4.4	59.0	0.7	61.3	1.8	48.8	0.3
24h Intact vs decapitated	0.0	97.3	0.4	93.3	1.5	91.5	0.0	96.3

B

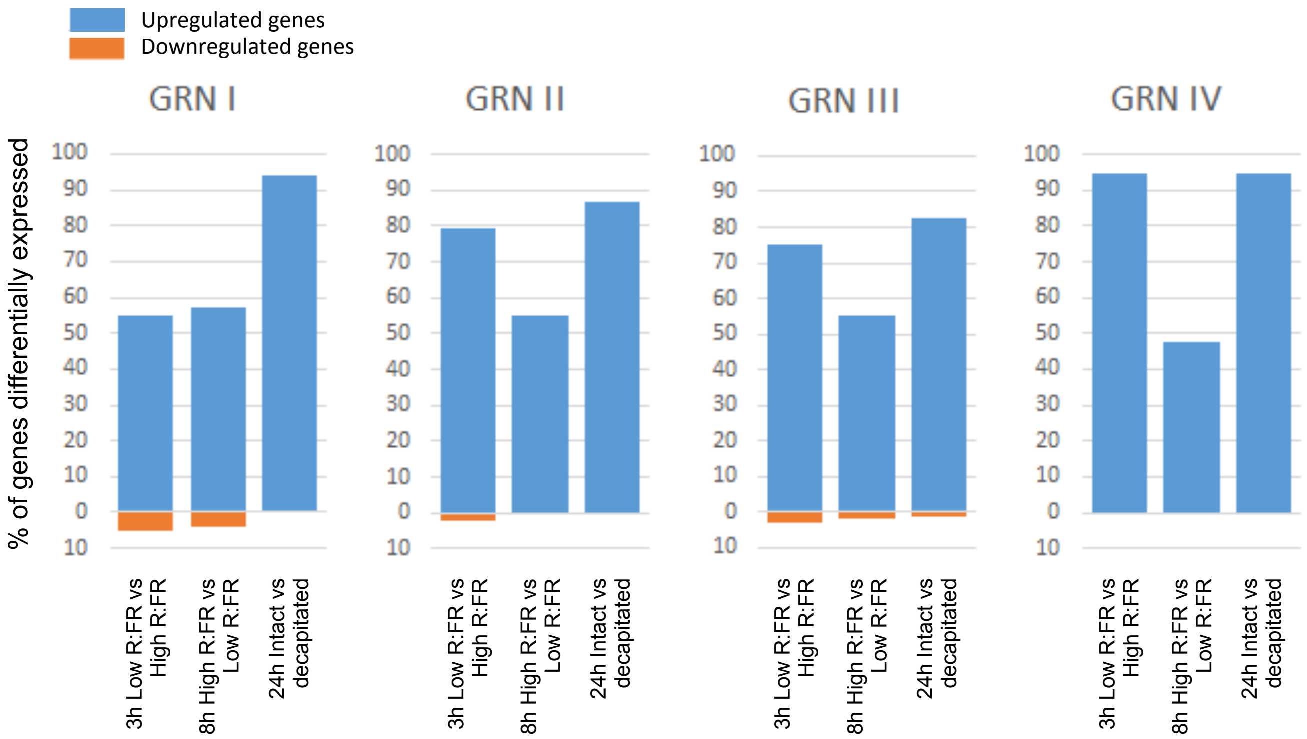

Figure S2. Transcriptomic response of Bud Dormancy genes and their coregulated genes in arabidopsis buds. A. Number of genes up- (FC ≥ 1.2) and down-regulated (FC ≤ -1.2) in dormant buds samples in each experiment. B. Graphical representation of the data in A. All the global responses are different from those expected at random (Chi-square test, p-value < 0.05).

GRN	I	II	III	IV
I	297	14	45	78
II	14	283	93	74
III	45	93	271	84
IV	78	74	84	295

GRN (%)	I	II	III	IV
I	-	4,71	15,15	26,26
II	4,95	-	32,86	26,15
III	16,61	34,32	-	31,00
IV	26,44	25,08	28,47	-

Figure S3. Degree of overlap between GRNs studied. Top, number of genes common for each pair of GRNs. Bottom, percentage of overlap between pairs of GRNs.

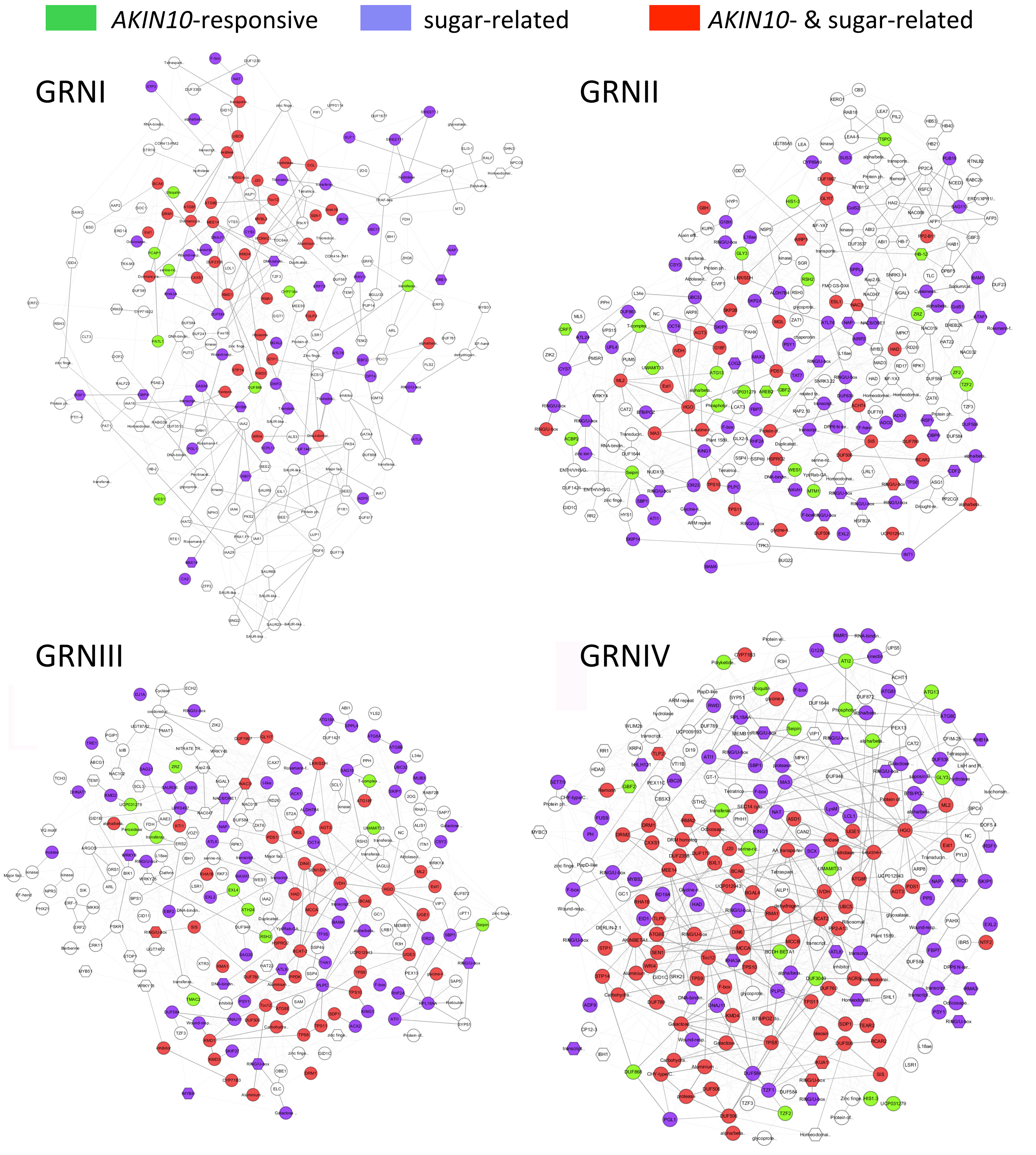

Figure S4. Bud dormancy GRNs. Cytoscape representation of genes within each GRN, according to ATTED-II Network Drawer (Obayashi et al 2007). Genes of unknown function have been omitted. In green, *AKIN10*-responsive genes (Baena et al. 2007). In purple, sugar-related genes. In red, sugar-related & *AKIN10*-responsive genes.

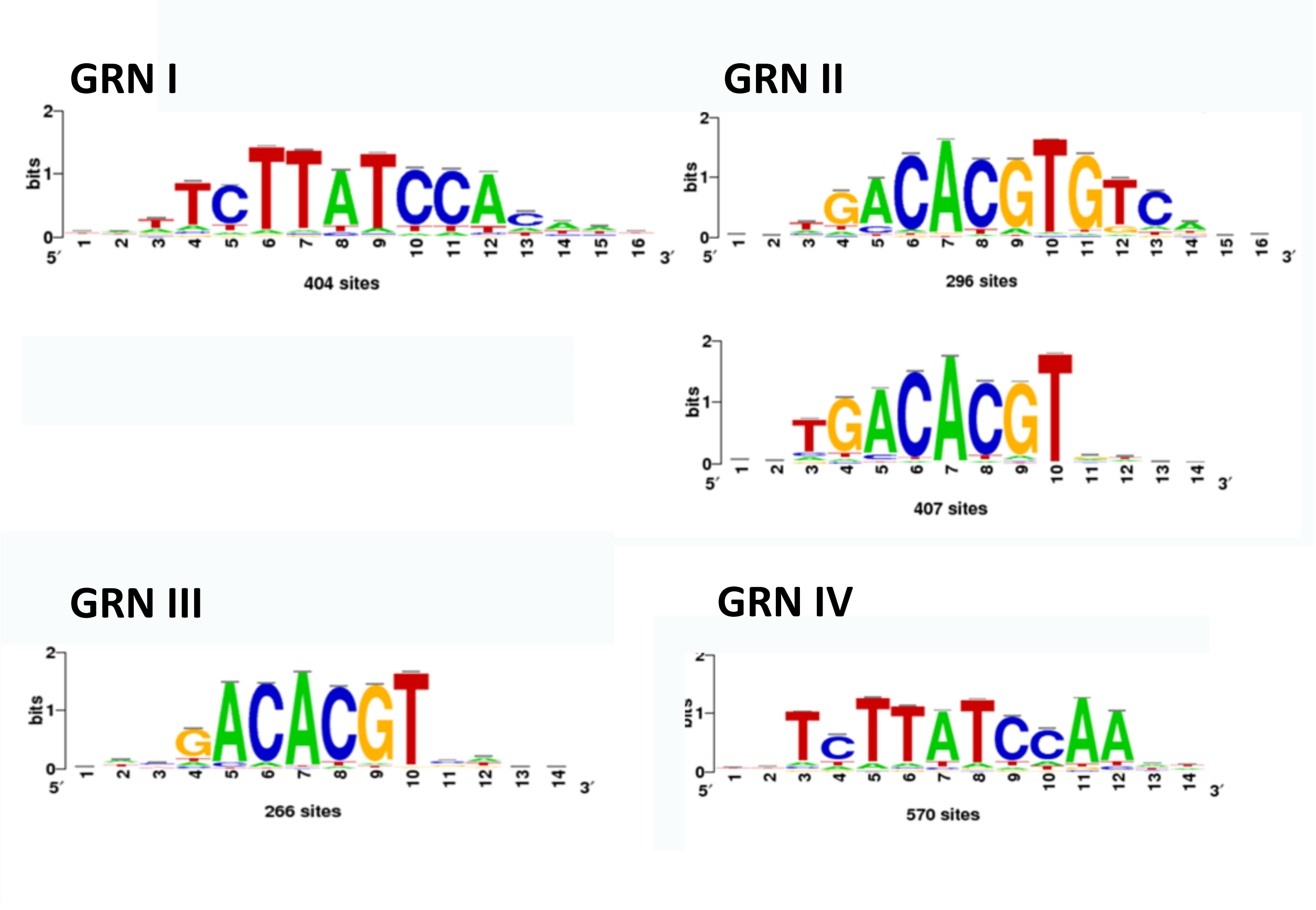

Figure S5. Regulation of bud dormancy GRNs. Most overrepresented motifs found in 1 kb genomic sequence upstream of the ATG of bud dormancy genes obtained with Oligo-analysis and Pattern-assembly using the default parameters (Rsat, Medina-Rivera et al. 2015). Matrices were converted into consensus motifs with Convert-matrix and represented using WebLogo (Crooks et al., 2004). The total number of sites found in each GRN is indicated below. Other overrepresenated motifs are in Dataset S4.

A

GRNI	GRNII	GRNIII	GRNIV
MYBS2			MYBS2, MYBH, At1g19000, At1g74840
	GBF2, GBF3		
	ABF3, ABF4		
	NAP, NAC6, ATAF1, ATAF1, NAC047, NAC3	NAP, NAC6, NAC047, NAC3, NAC102, RD26	

B

TF			Number of binding sites in the genome		Number of binding sites in the GRN		
AGI	Name	GRN	N	Frequency	N Expected	N Observed	p-value
AT5G08520	MYBS2	I	4133	0.123	36.5	80	<0.001
AT5G08520	MYBS2	IV	4133	0.123	36.3	95	<0.001
AT5G47390	MYBH/KUA1	IV	12035	0.358	105.7	182	<0.001
AT1G19000	MYB-related	IV	9687	0.288	85.0	156	<0.001
AT1G74840	MYB-related	IV	7603	0.226	66.7	131	<0.001
AT2G46270	GBF3	II	14334	0.427	120.7	243	<0.001
AT4G01120	GBF2	II	10938	0.326	92.1	229	<0.001
AT4G34000	ABF3	II	10150	0.302	85.5	229	<0.001
AT3G19290	ABF4	II	7168	0.213	60.4	205	<0.001
AT1G69490	NAP	II	4061	0.121	34.2	90	<0.001
AT1G69490	NAP	III	4061	0.121	32.8	91	<0.001
AT5G39610	NAC6/ORE1	II	2466	0.073	20.8	40	<0.001
AT5G39610	NAC6/ORE1	III	2466	0.073	19.9	40	<0.001
AT1G01720	ATAF1	II	7615	0.227	64.1	136	<0.001
AT3G04070	NAC047	II	6448	0.192	54.3	132	<0.001
AT3G04070	NAC047	III	6448	0.192	52.0	129	<0.001
AT3G15500	NAC3/NAC55	II	6370	0.190	53.6	123	<0.001
AT3G15500	NAC3/NAC55	III	6370	0.190	51.4	128	<0.001
AT4G27410	NAC72/RD26	III	8384	0.250	67.6	217	<0.001
AT5G63790	NAC102	III	3182	0.095	25.7	150	<0.001

Figure S6. Regulation of bud dormancy GRNs (cont). A. Transcription factors in each GRN that could potentially bind the consensus motif B. Number and frequency of genes bound by each potential GRN master regulator (TF) in the Arabidopsis genome (33602 genes). Number of expected and observed binding sites in each GRN. All values are significantly higher than those expected in a random gene list (Pearson´s test). White background indicates values from Dap-Seq data (O´Malley et al. 2016), grey background from ChIP-Seq data (Song et al., 2016).

A

Populus	1WSD/LD		2WSD/LD		3WSD/LD		4WSD/LD		5WSD/LD		6WSD/LD	
GRN	N up	N down	Nup	N down	N up	N down	N up	N down	N up	N down	N up	N down
I	69	37	66	38	62	42	66	40	65	40	66	40
II	111	45	122	35	126	32	131	27	133	26	128	31
III	86	40	96	29	92	32	99	27	100	25	102	24
IV	117	40	123	34	119	40	125	34	129	29	119	40
	% up	% down	% up	% down	% up	% down	% up	% down	% up	% down	% up	% down
I	65.1	34.9	62.3	35.8	58.5	39.6	62.3	37.7	61.3	37.7	62.3	37.7
II	69.8	28.3	76.7	22.0	79.2	20.1	82.4	17.0	83.6	16.4	80.5	19.5
III	68.3	31.7	76.2	23.0	73.0	25.4	78.6	21.4	79.4	19.8	81.0	19.0
IV	73.6	25.2	77.4	21.4	74.8	25.2	78.6	21.4	81.1	18.2	74.8	25.2

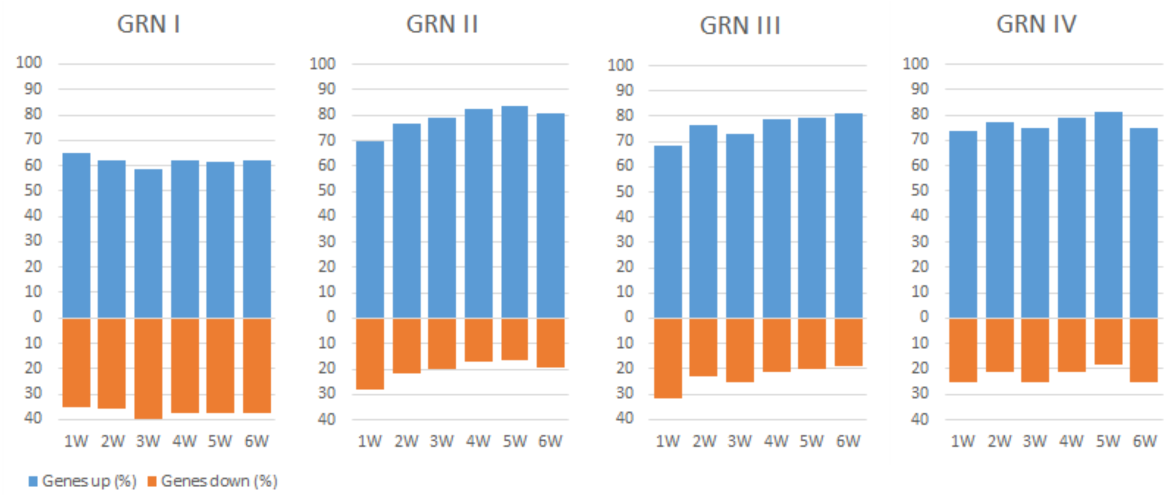

B

Vitis	JUL/APR		SEP/APR		NOV/APR		JAN/APR		MAR/APR	
GRN	N up	N down	N up	N down	N up	N down	N up	N down	Nup	N down
I	81	42	83	40	80	42	89	34	85	38
II	137	19	131	24	131	23	135	20	137	19
III	109	28	106	30	101	35	111	26	113	24
IV	133	35	134	35	136	34	140	29	134	36
	% up	% down	% up	% down	% up	% down	% up	% down	% up	% down
I	65.9	34.1	67.5	32.5	65.0	34.1	72.4	27.6	69.1	30.9
II	87.8	12.2	84.0	15.4	84.0	14.7	86.5	12.8	87.8	12.2
III	79.6	20.4	77.4	21.9	73.7	25.5	81.0	19.0	82.5	17.5
IV	78.2	20.6	78.8	20.6	80.0	20.0	82.4	17.1	78.8	21.2

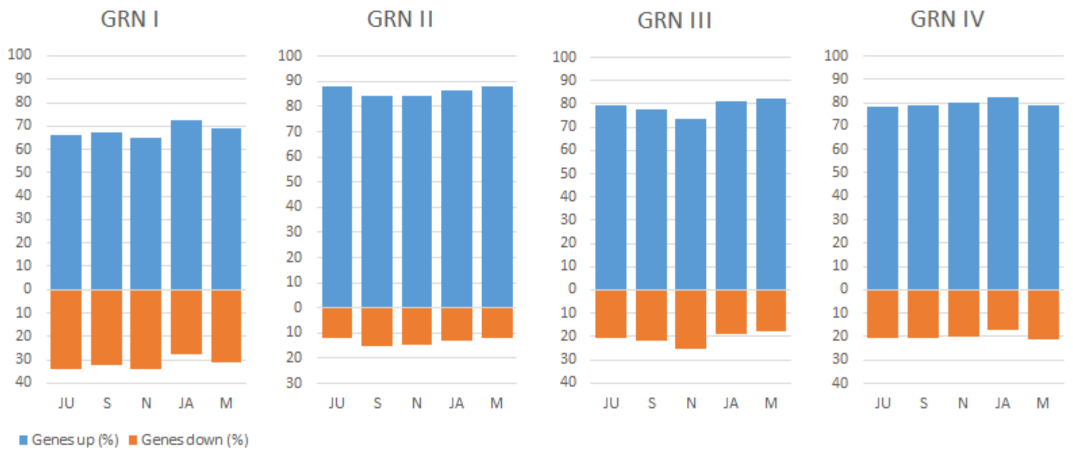

Figure S7. Transcriptomic response of Bud Dormancy genes and their coregulated genes in poplar apical buds (A) and grapevine axillary buds (B). Top, Number of genes up- and down-regulated in each sample relative to the "active bud" sample"sample. B.Graphical representation of the data in A. All the responses are different from those expected at random (Chi-square test, p-value < 0.05).

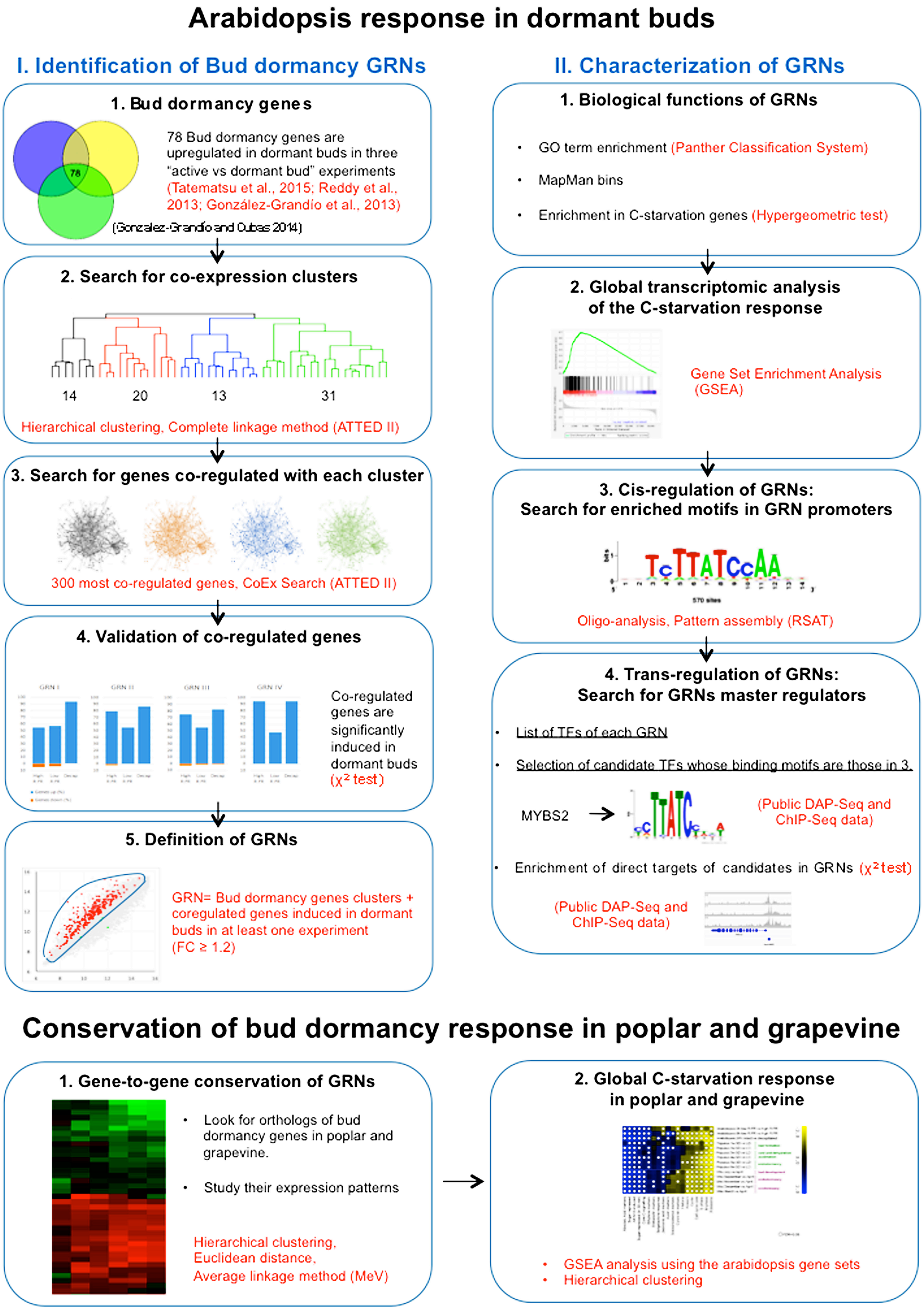

Figure S8. Summary of the workflow carried out in this work.

Arabidopsis gene code	Name	Annotation in PGSC v4.03 pseudomolecules	
At1g28330	DYL1	Auxin-repressed protein	PGSC0003DMT400033385
At5g27320	GIDC1	Gibberellin receptor GID1	PGSC0003DMT400073501
At2g18050	HIS1-3	Histone H1	PGSC0003DMT400009344
At1g69490	NAP gene a	NAC domain protein NAC2	PGSC0003DMT400047440
At1g69490	NAP gene b	NAC domain protein NAC2	PGSC0003DMT400007341

Figure S9. Identifiers of potato orthologs analyzed

>qStDYL1_F	GATCCAAGCATCAGTGAAATGAAA
>qStDYL1_R	GCTCTGTCACGATCCAAAATCTCT
>qStEXL2_F	GGTAGGTCGGTGAACTTAGTGTTGA
>qStEXL2_R	AAATCCTTCCACGAATACATCCTTC
>qStGBF3_F	TCAACTGATGGAAGTGACACAAATG
>qStGBF3_R	TTTCTTACTTCTCTCACTGACCCCA
>qStGID1C_F	GTGTCTATCGCCCTTCTTTTGAG
>qStGID1C_R	GTTCAATTATACTCGGTGAAGCTCC
>qStHIS1-3_F	CTAATCAAAATCAAGGCTTCATACAAA
>qStHIS1-3_R	GTAGTCTCCTTCTTTCCAGCCTCAG
>qStNAPa_F	AATTGGAGGCACAAAATGTGAGTAT
>qStNAPa_R	CACCAACAACTTCAATTGCATTATT
>qStNAPb_F	GACCAATGGATATGTTGTGCCTC
>qStNAPb_R	CCCTAGTTGAAATATCTCCATGGCT

Table SI. Primers used for qRT-PCR (5´-3´)
